# Synthesis, Photochemistry, Computational Study and Potential Application of New Styryl-Thiophene and Naphtho-Thiophene Benzylamines

**DOI:** 10.3390/ijms24010610

**Published:** 2022-12-29

**Authors:** Milena Mlakić, Ilijana Odak, Ivan Faraho, Martina Bosnar, Mihailo Banjanac, Zlata Lasić, Željko Marinić, Danijela Barić, Irena Škorić

**Affiliations:** 1 Department of Organic Chemistry, Faculty of Chemical Engineering and Technology, University of Zagreb, Marulićev trg 19, HR-10000 Zagreb, Croatia; 2 Department of Chemistry, Faculty of Science and Education, University of Mostar, Matice Hrvatske bb, 88000 Mostar, Bosnia and Herzegovina; 3 Pharmacology In Vitro, Selvita Ltd., Prilaz Baruna Filipovića 29, HR-10000 Zagreb, Croatia; 4 Teva api Analytical R&D, Pliva, Prilaz Baruna Filipovića 25, HR-10000 Zagreb, Croatia; 5 NMR Center, Ruđer Bošković Institute, Bijenička Cesta 54, HR-10000 Zagreb, Croatia; 6 Group for Computational Life Sciences, Division of Physical Chemistry, Ruđer Bošković Institute, Bijenička Cesta 54, HR-10000 Zagreb, Croatia

**Keywords:** acid resistance, benzylamines, DFT, heterostilbenes, molecular docking, photocyclization, semiempirical methods, TD-DFT

## Abstract

In this research, the synthesis, photochemistry, and computational study of new *cis*- and *trans*-isomers of amino-thienostilbenes is performed to test the efficiency of their production and acid resistance, and to investigate their electronic structure, photoreactivity, photophysical characteristics, and potential biological activity. The electronic structure and conformations of synthesized thienostilbene amines and their photocyclization products are examined computationally, along with molecular modeling of amines possessing two thiophene rings that showed inhibitory potential toward cholinesterases. New amino-styryl thiophenes, with favorable photophysical properties and proven acid resistance, represent model compounds for their water-soluble ammonium salts as potential styryl optical dyes. The comparison with organic dyes possessing a *trans*-aminostilbene subunit as the scaffold shows that the newly synthesized *trans*-aminostilbenes have very similar absorbance wavelengths. Furthermore, their functionalized *cis*-isomers and photocyclization products are good candidates for cholinesterase inhibitors because of the structural similarity of the molecular skeleton to some already proven bioactive derivatives.

## 1. Introduction

Stilbene derivatives have been a focus of scientific interest over the years as model compounds in the fundamental investigation of their photoisomerization reaction [1,2,3,4] and the application of these molecules in materials science [5]. Heteroaromatic derivatives of the stilbene molecules (heterostilbenes) have shown important properties for use in many attractive fields [6,7]. The investigation of the photoreactivity of heterostilbenes showed potential applications in optoelectronics [8], as well as starting materials for photochemical reactions [9,10]. Photophysical properties of furo- and thienostilbenes were investigated [11], and heterostilbenes have found application in so-called NLO materials (nonlinear optical materials) [12,13]. Such materials are crucial in quantum electronics, telecommunications technology [14], and nanotechnology [15,16]. Some authors have investigated the *E-Z* photoisomerization and electrocyclization of substituted phenylethenyl furans, thiophenes, and oxazoles [17,18,19]. Moreover, nitro-substituted heterostilbenes were especially interesting because of their fluorosolvatochromism and hyperpolarizability [20,21]. Amino-substituted stilbenes represent the basic skeleton of amino acid-stilbene quaternary ammonium salt optical dyes [22,23,24]. As functional optical materials, stilbene optical dyes represent the main body of fluorescent whitening agents. Stilbene optical dyes are usually applied only under neutral or weakly alkaline conditions because of their weak acid resistance. In contrast, the amino-substituted stilbene quaternary ammonium salts [22] with photophysical properties as optical dyes show strong acid resistance. Biological activity experiments in vitro proved that this kind of compound showed significant antibacterial activity against several types. On the other hand, the photocyclization reactions of heterostilbenes gave interesting planar substrate hetero-naphthalenes as starting material for potential intercalators synthesis [25]. To carry out the electrocyclization as successfully as possible, the photochemical cyclization mechanisms and the proof of intermediate dihydronaphthalenes are also studied in detail [26]. The key intermediate involved in the photocyclization of stilbene, dihydrophenanthrene, is known to be unstable; however, functionalized stilbenes (again) with amino groups greatly enhanced the stability of the intermediate, allowing quantitative isolation and full characterization of these rare species.

**Figure 1:**
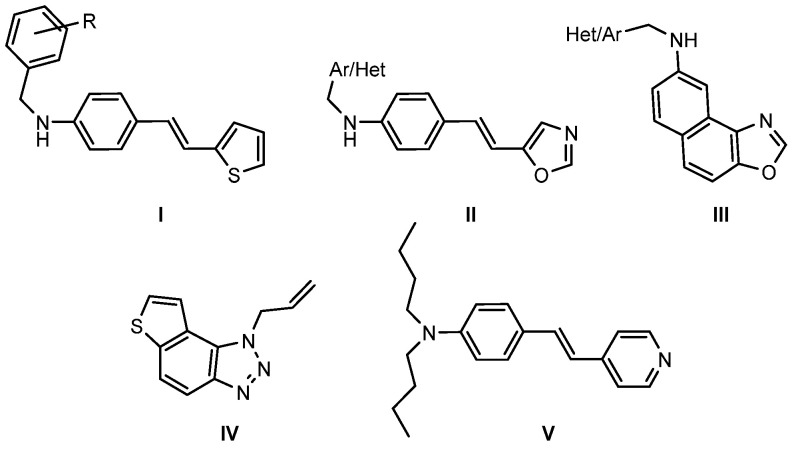
The basic structure of new and known heterostilbenes and their photocyclization products: new amino-thienostilbenes (**I**), amino-oxazolostilbenes (**II**) [27], naphthooxazolo-amines (**III**) [27], and thienobenzo-triazoles (**IV**) [29], as proven active compounds, and amino-styryl organic dye (**V**) [30].

According to our previous experience in the field of the synthesis and photochemistry of heterostilbene molecules [27,28,29], we herein designed, synthesized, and photochemically transformed the new amino-thienostilbenes (Figure 1I) as interesting structures with potential biological activity and specific chemical and photophysical characteristics.

In our previous paper [27], new *trans-*amino-4-/5-arylethenyl-oxazoles (Figure 1II) were designed and synthesized. In addition, they were transformed into naphthoxazole benzylamine photoproducts (Figure 1III) by an efficient photocyclization reaction (Figure 1). Among these new compounds, naphthoxazole benzylamines (Figure 1III) stood out as potential cholinesterase inhibitors, butyryl- (BChE) over acetylcholinesterase (AChE), with IC_50_ values in µM range. 

An additional motivation for the design of new heterostilbenes and their photocyclization products was provided by the results of biological testing of 1,2,3-triazolo(thieno)stilbenes and their photoproducts thienobenzo/naphtho-triazoles, which showed BChE inhibition associated with the inhibition of TNF*α* cytokine production and anti-inflammatory activity. The best experimental results were achieved with the allyl-thienobenzotriazole (Figure 1IV), which is twice as potent an inhibitor of eqBChE as compared to the standard galantamine. At the same time, this compound strongly inhibited TNFα production in PBMCs in response to the LPS stimulus. The use of heterostilbenes as styryl dyes for fluorescence tests for biomedical research is also common [30,31]; those with a styryl skeleton often contain an amino group (Figure 1V). Cationic styryl dyes show a high affinity for binding to various types of DNA and high cytotoxicity in tests with cancer cells [32]. Interest in the research of synthetic dyes stems from their chemical and physical properties, such as light emission, photochemical activity, and photosensitization. Styryl dyes can be used as optical recording media, sensitizers, and laser dyes [33].

In this research, based on all the above knowledge, the synthesis, photochemistry, and computational study of new amino-thienostilbenes were performed to investigate their electronic structure, photoreactivity, photophysical characteristics, and potential biological activity—at this stage without more serious functionalization. The electronic structure and conformations of synthesized compounds are examined computationally, along with molecular modeling of compounds that showed inhibitory potential toward cholinesterases. New amino-thienostilbenes, stable under acidic conditions and with favorable photophysical properties, could be used to prepare their water-soluble ammonium salts as potentially biologically active compounds. On the other hand, their functionalized photocyclization products could be good candidates for cholinesterase inhibition, given that they have the same basic skeleton as some already proven bioactive derivatives [28,29]. 

## 2. Results and Discussion

### 2.1. Synthesis, Photophysical Properties, and Photochemistry of Styryl-Thiophene Benzylamines 2–8 toward Photocyclization Products 9–15

**Scheme 1:**
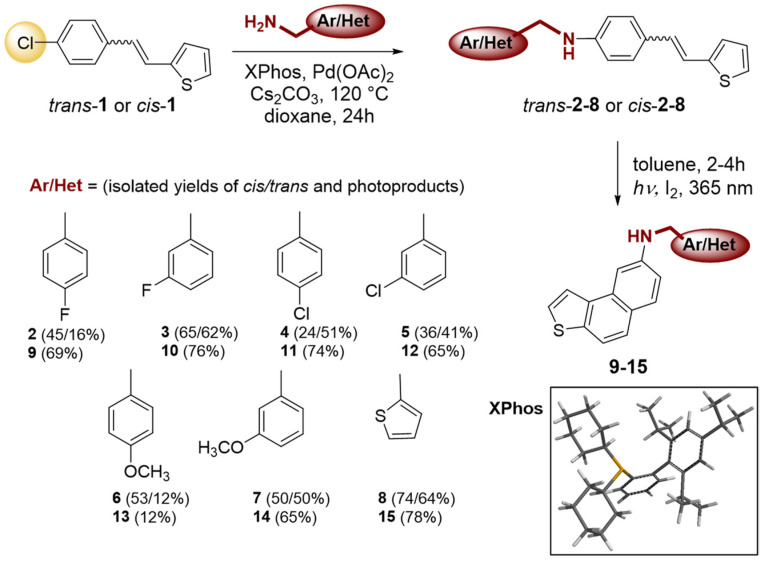
Synthesis and photocyclization reactions of styryl-thiophene benzylamines **2**–**8**. (For compound **7**, the percentages do not mean isolated yields but the relative ratio of the obtained configurational isomers, given that the reaction photo-mixture also contains aldehyde as a minor byproduct.).

New styryl-thiophene benzylamines **2**–**8** (Scheme 1) are synthesized by the Buchwald-Hartwig amination reactions [27] from the previously prepared chloro derivative **1** [34]. The aim was to synthesize new amines possessing the specific stilbene-based skeleton. The amination of pure *trans*-**1** or *cis*-**1** using XPhos, Pd(OAc)_2_ in dioxane, Cs_2_CO_3_, and different benzylamines at 120 °C successfully gave isolated new amines **2**–**8** (Scheme 1) in 24–74% yields for *cis*-, and in 12–64% yields as *trans*-isomers. A closer look into the nature and position of the substituent on the isolated yield (Scheme 1) reveals that the *m*-fluoro (*cis*-**3**) and thienyl (*cis*-**8**) derivatives give the best yields in the case of *cis*-configuration, as well as of *trans*-amines. Based on our previous results [35], where the *ortho* substitution on the arylamines gave the lowest yields, we decided to exclude this kind of starting amine at this point of the investigation. However, the *ortho* substitutions usually result in compounds with some unique properties; therefore, they will be considered and studied in separate research.

**Figure 2:**
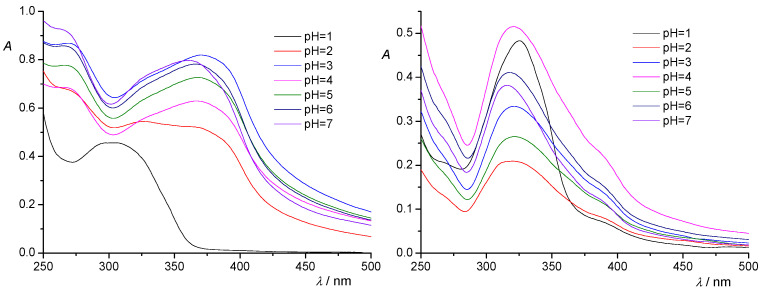
Changes in the UV spectra of compound *trans*-**3** (**left**) and *trans*-**4** (**right**) as a function of pH in buffer aqueous solution for pH from 7 to 1.

**Figure 3:**
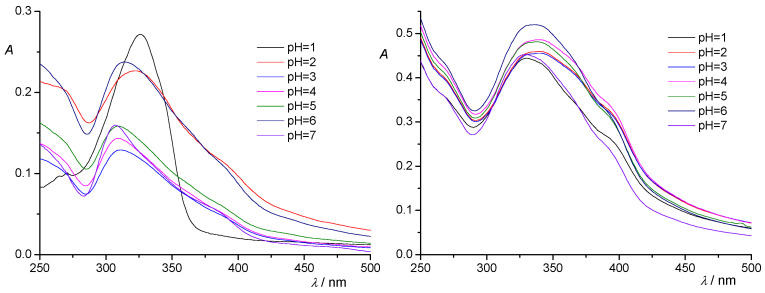
Changes in the UV spectra of compound *cis*-**8** (**left**) and *trans*-**8** (**right**) as a function of pH in buffer aqueous solution for pH from 7 to 1.

To analyze the acid resistance of the synthesized amino-thienostilbenes **2**–**8** for their potential applications, the pH change of the UV spectra was recorded for compounds *trans*-**3**, *trans*-**4**, *cis*-**8,** and *trans*-**8,** as shown in Figure 2 and Figure 3. pH changes were recorded in the buffer aqueous solution for pH 7 to 1. Compounds were previously dissolved in methanol p.a. at the concentration 2 *×* 10*^−^*^3^ mol dm*^−^*^3^, followed by the concentration adjustment in the buffer solution to 2 × 10*^−^*^5^ mol dm*^−^*^3^. However, it is known that because of the presence of the double bond, stilbene optical dyes generally possess poor light resistance. Under the action of light, they easily isomerize, resulting in decreased fluorescent activity and a poor-lasting application effect. As already mentioned, the amino-substituted stilbene quaternary ammonium salts [22] with photophysical properties as optical dyes showed strong acid resistance. Compared to them, the new amino-thienostilbenes **2**–**8** are also stable under acid conditions, except at pH 1 for the only analyzed *cis*-isomer of compound **8**. All *trans*-isomers showed only slight changes in the UV maxima in the range from pH 7 to 2, and a more significant change occurred only at pH 1 (Figure 2 and Figure 3). The strong absorption of *trans*-**3** shifted from ~380 nm to ~310 nm when pH changed to 1 (Figure 2). Furthermore, at pH 1, the bathochromic shift is visible for *cis*-**8**, suggesting possible isomerization to *trans*-**8**. As the chemical shifts of ethylenic protons for *cis*-**8** and *trans*-**8** are quite different, the NMR analysis as a control experiment was provided for the *cis*-**8** at pH 1 to confirm the predicted isomerization and to additionally investigate the acid resistance of non-quaternized stilbenes **2**–**8** as model compounds. In the ^1^H NMR spectrum of *cis*-**8** recorded at pH 1, the coupling constant typical for cis configuration is still present, confirming the strong acid resistance of this compound. However, signals are slightly shifted because of the protonation of the amino group.

**Figure 4:**
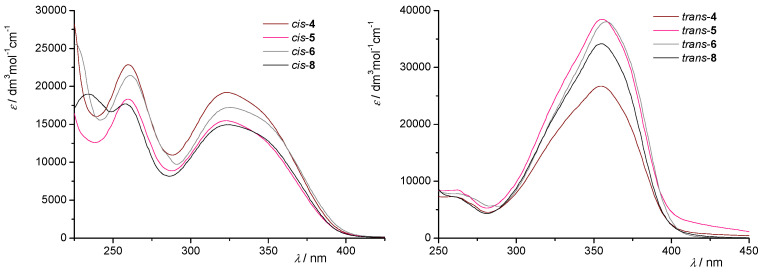
Representative UV-Vis spectra (ACN) of *cis*- and *trans*-isomers of newly synthesized styryl-amines **4**–**6** and **8**.

The further intention of the research was to transform the synthesized styryl-thiophene benzylamines **2**–**8** into their photocyclization products, naphthothiophene benzylamines **9**–**15** (Scheme 1), compounds with interesting physico-chemical properties that would also be examined for their potential biological activity. In aerobic conditions, a mixture of isomers of **2**–**8** was dissolved in toluene (~2.5 × 10*^−^*^3^ M) and irradiated with 10 UV lamps at 365 nm in a quartz vessel with the addition of a catalytic amount of iodine in a photochemical reactor Rayonet for 2–4 h to achieve almost complete conversion. The wavelength of 365 nm was chosen based on UV-Vis spectra of several *cis*- and *trans*-isomers of styryl-amines (Figure 4), which show that their spectra are typical for diarylethene [9,10] with a strong absorption maximum at about 355 nm for the *trans*-isomers and less intense and hypsochromically shifted bands for the *cis*-isomers (see Experimental Section). The UV-Vis spectra of new amines **2**–**8** also agree with the recorded spectra of several optical dyes because of their structural similarity (aminostilbene skeleton). 

**Figure 5:**
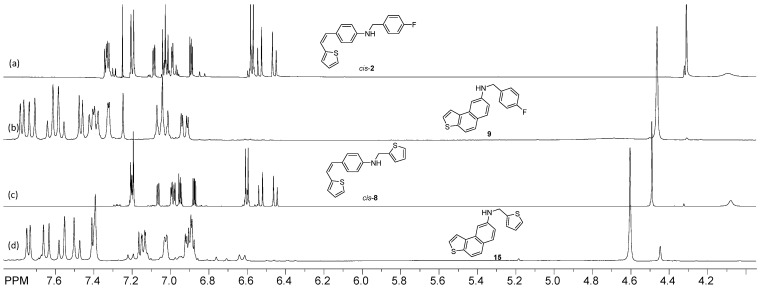
Selected ^1^H NMR spectra (CDCl_3_) of *cis*-**2** (**a**) and *cis*-**8** (**c**) and their photocyclization products **9** (**b**) and **15** (**d**), respectively.

Preparative irradiations of **2**–**8** gave the naphthothiophene amines **9**–**15** in 65–78% of isolated yields (see Scheme 1 and Experimental Section), except for the *para*-methoxy benzyl derivative **13** (12% of isolated yields). This is not surprising, as *para*-methoxy benzyl is often used as a photolabile protecting group (PPG). PPGs are used as protecting groups that can be easily removed by photoirradiation under mild conditions. Some other benzyl groups also serve as photolabile protecting groups, but methoxy-substituted benzyls are among the most labile, giving aldehyde as a byproduct after cleavage. Both heterostilbenes **6** and **7** with methoxy substituents in *meta*- and *para*-position showed in the NMR spectra of their photoproducts **13** and **14** signals for the presence of aldehydes (see ESI). The amino-thienostilbenes **2**–**8** and their photoproducts **9**–**15** were completely characterized by ^1^H and ^13^C NMR spectroscopy and HRMS analyses (Figure 5, Experimental and ESI, Figures S1–S79). The formation of the photoproducts **9**–**15** was generally accompanied by the appearance of some high-molecular-weight products, which were not investigated. In the ^1^H NMR spectra of **9**–**15**, the ethylenic protons’ disappearance and loss of one signal of the thiophene ring was observed, compared with the starting triazolo(thieno)stilbenes **2**–**8**. The NH protons in new amines **2**–**15** appear in the aliphatic region between 4.1 and 5.0 ppm. In addition, the characteristic new signal for the methylene group of the benzyl moiety was detected for all amines **2**–**15** (see ESI and Experimental Section). 

### 2.2. Computational Study of the Electronic Structure of Styryl-Thiophene and Naphtho-Thiophene Benzylamines 2–15

**Figure 6:**
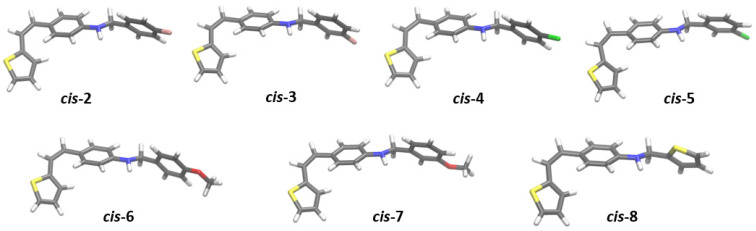
The most stable conformers of *cis*-isomers of styryl-thiophene benzylamines **2**–**8**, optimized at the M06-2X/6-31g(d) level of theory.

Structures of all examined molecules (**2**–**15**) were optimized at the M06-2X/6-31G(d) level of theory. Examination of the conformational space of *cis*-isomers of styryl-thiophene benzylamines **2**–**8** showed that substituents at benzyl moiety did not impact the stability of the scaffold conformation. This result was not surprising given the similarities in their experimentally obtained UV-Vis spectra. Figure 6 shows the most stable conformers of *cis*-isomers of compounds **2**–**8**.

To get additional insight into the experimental data, we calculated the UV-Vis spectra of all compounds using time-dependent density functional theory (TD-DFT). A time-dependent perturbation equation based on the Runge–Gross theorems [36] was solved for 20 excited singlet states for each optimized molecule, using the CAM-B3LYP/6-31++G(d) level of theory, with a conductor-like polarizable continuum model (CPCM) for a description of the solvent acetonitrile (ACN). Experimental and calculated data for *cis*-isomers of styryl thiophene benzylamines are given in Table 1, along with the main assignments predicted by calculations. 

**Figure 7:**
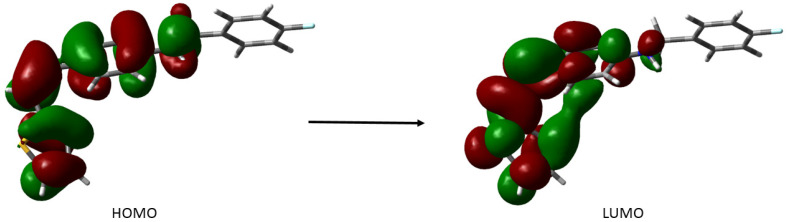
Canonical molecular orbitals involved in transition in UV-Vis spectra of compound *cis*-**2**.

The spectra of all *cis*-styryl-thiophene benzylamines are mutually very similar; the position of absorption maxima is at ~325 nm. The calculated *λ*_max_ values for these compounds agree with experimental data, assigning this absorption maximum to the transition from the highest occupied molecular orbital (HOMO) to the lowest unoccupied one (LUMO). The canonical molecular orbitals involved in this transition for molecule *cis*-**2** are presented in Figure 7. Expectedly, π-electrons of the substituted phenyl belonging to the benzylamine part do not participate in the transition because of the presence of sp^3^ carbon that impedes the efficient conjugation with the π-system of the rest of the molecule. The same holds for other *cis*-styryl-thiophene benzylamines.

**Figure 8:**
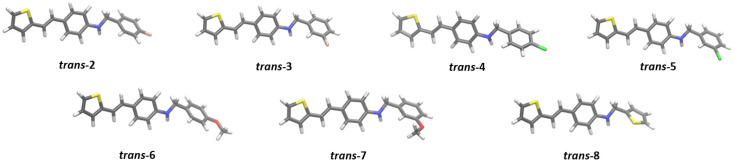
The most stable conformers of *trans*-isomers of styryl-thiophene benzylamines **2**–**8**, optimized at the M06-2X/6-31g(d) level of theory.

The most stable conformers of *trans*-isomers of styryl-thiophene benzylamines are presented in Figure 8. 

According to Table 2, their UV-Vis spectra also do not differ from each other, being consistently higher compared to their *cis*-counterparts. The values of the experimentally obtained *λ*_max_ are ~355 nm; calculations, however, predict slightly lower absorption maxima values of ~340 nm. Just as for *cis*-isomers, in all spectra of *trans*-isomers, the absorption maximum is assigned to the HOMO-LUMO transition.

**Figure 9:**
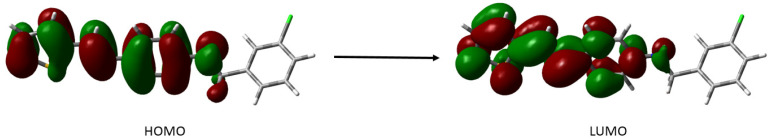
Canonical molecular orbitals involved in transition in UV-Vis spectra of compound *trans*-**5**.

The canonical orbitals presented in Figure 9 illustrate that, because of the lack of conjugation with the styryl-thiophene scaffold, π-electrons belonging to benzylamine are not involved in the transition responsible for the absorption maximum found in the spectra of trans-isomers. 

**Figure 10:**
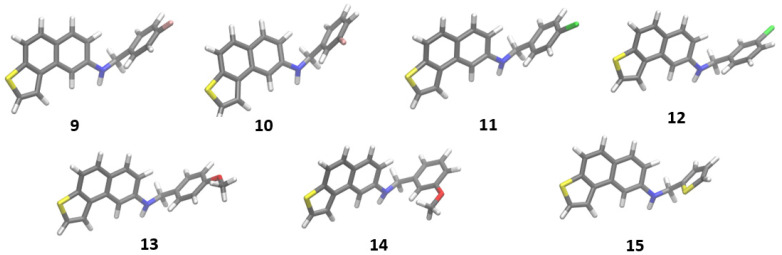
The most stable conformers of *trans*-isomers of naphtho-thiophene benzylamines **9**–**15**, optimized at the M06-2X/6-31g(d) level of theory.

Figure 10 shows optimized structures of naphtho-thiophene benzylamines **9**–**15**. Their UV-Vis spectra reveal a more complicated situation, and calculations did not correctly reproduce their complexity. Namely, all calculated spectra predict three peaks: at ~310, 238, and 220 nm, assigning them to transitions HOMO → LUMO, HOMO − 1 → LUMO, and HOMO − 2 → LUMO, respectively (Table S1). Measured data show much more diversity: peak corresponding to the H → L transition is not observed in compounds **11** and **12**. A certain agreement between computationally predicted data and experiment is found only for molecules **13** and **15**, where two additional peaks (at 255 and 245 nm) are present; however, calculations underestimated these maxima for 20–25 nm. Detailed results are shown in Table S1.

**Figure 11:**
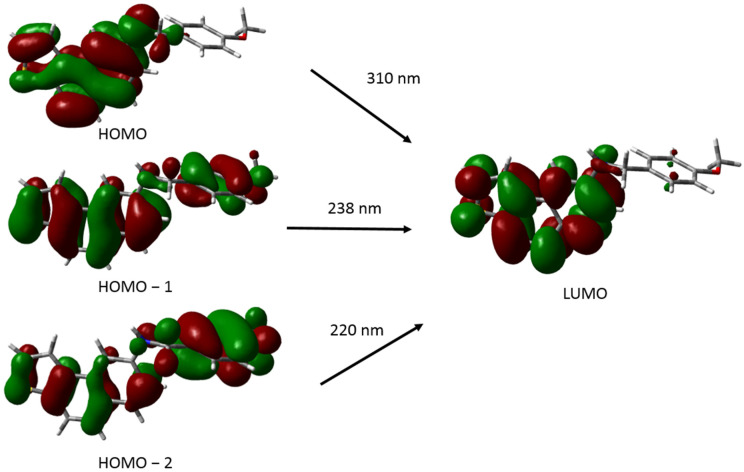
Canonical molecular orbitals involved in transitions in UV-Vis spectra of compound **13**.

The canonical orbitals that participate in assigned transitions for molecule **13** are presented in Figure 11. The involvement of HOMO − 1 and HOMO − 2 in transitions occurs because of the partial conjugation between the π system of the naphtho-thiophene scaffold and π electrons of the benzylamine moiety.

### 2.3. Biological Potential of Styryl-Thiophene and Naphtho-Thiophene Benzylamines 2–15

Continuing our previous research on triazolo-stilbenes and triazolo-thienostilbenes [28], new styryl-thiophene and naphtho-thiophene benzylamines **2**–**15** were tested for acetyl- and butyrylcholinesterase inhibition efficacy. Commercially available galantamine was used as a reference standard. Among all the tested styryl-thiophene and naphtho-thiophene benzylamines, only two compounds inhibited ChEs in the μM range, *cis*-**8** and **15**. Compound *cis*-**8**, which belongs to amino-thienostilbenes, showed the inhibitory potential toward AChE, with an IC_50_ value of 132.6 μM (IC_50_ value for AChE for reference standard galantamine is 0.15 μM). Other amino-thienostilbenes, *cis*-**2**–**6**, *trans*-**2**–**6,** and *trans*-**8,** achieved inhibition values smaller than 50%, regardless of the tested enzyme. Electrocyclic derivative **15** showed the most promising inhibitory potential, with an IC_50_ value of about 32.3 μM for BChE (IC_50_ of galantamine is 7.9 μM for BChE). The achieved inhibitory activity of compound **15** toward BChE is in the range of triazolo-stilbenes, triazolo-thienostilbenes, and their photocyclization products that were previously investigated [28]. In that paper, it was found that the triazolo-thienostilbene derivative with *trans*-configuration and propyl substituent at 1,2,3-triazolo ring inhibited BChE with an IC_50_ value of 18.7 μM [28], whereas its photocyclization product was active toward both enzymes (IC_50_ 48.8 μM for BChE and 607.0 μM for AChE). Among these derivatives, the best result was achieved with allyl-thienobenzotriazole **IV** (Figure 1), which inhibited BChE with an excellent IC_50_ value of 3.8 μM and AChE with a very good IC_50_ value of 56.2 μM. In the research we present here, two compounds, *cis-***8** and its photoproduct **15**, are also comparable to the amino-oxazolostilbenes **II** (Figure 1) that we previously studied [27]. It turns out that the introduction of the second thiophene ring increases activity toward cholinesterase inhibition in comparison with those possessing only one thiophene. Some other studies have also found that replacing benzene with a heteroaromatic ring improves biological activity in this context [37,38].

Testing the potential inhibitory effect of the styryl-thiophene and naphtho-thiophene benzylamines toward cholinesterases gave promising results for compounds *cis*-**8** with AChE and its photoproduct **15** with BChE. Notably, both molecules have a second thiophene ring instead of substituted benzyl. Therefore, we performed molecular docking of these molecules to elucidate the structure of the complex between ligand and enzyme and possible interactions between tested compounds and the enzyme. Molecular docking was performed using the Autodock program package [39]. The most stable structures of the complex between compounds and cholinesterase obtained by the docking were additionally optimized using a semi-empirical approach, as described in the Computational Details section. 

**Figure 12:**
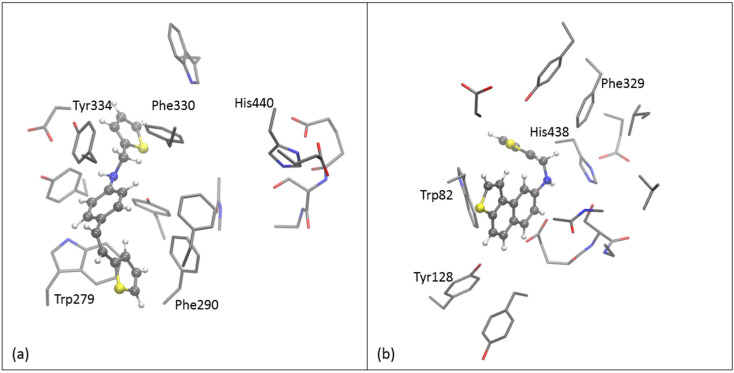
The optimized complexes between the active sites of cholinesterases and tested ligands: (**a**) compound *cis*-**8** and AChE, and (**b**) compound **15** and BChE.

The structures presented in Figure 12 reveal that π-π stacking is the main stabilizing interaction between these two ligands and the active site residues. The structure of the complex between *cis*-8 and the active site of AChE shows that the thiophene ring linked to the double CC bond is sandwiched between the peripheral anionic site and acyl pocket, interacting with Trp279 and Phe290. The second thiophene ring is placed conveniently in the proximity of Phe330 (at a distance of ~5 Å), while tyrosines surround the central aromatic ring. The ligand **15** acts similarly within the active site of BChE: the naphthalene core with annelated thiophene ring interacts with Trp82 and Tyr118, being placed at 4.0 and 4.9 Å from them. The second thiophene is stabilized by π-π stacking with Phe329, His438, and Trp82. 

Newly synthesized styryl-thiophene and naphtho-thiophene benzylamines (**2**–**15**) were also evaluated for biological activity in three different assays. First, the potential anti-inflammatory activity was tested by measuring TNFα production in human PBMCs following compound treatment and LPS stimulation. The structures *trans*-**2**, **9,** and **13** did not have any effect on TNFα production, while the rest of the tested compounds further increased the production of this cytokine upon stimulation. Since only *cis*-**8** and its photoproduct **15** showed moderate inhibitory potential toward ChE in biochemical assays, it was expected that our compounds would not have anti-inflammatory activity, which makes this observed enhancement of cytokine production an interesting discovery. Furthermore, the acute monocytic leukemia cell line THP-1 was treated with compounds for up to 72 h to test whether the compounds exerted any antiproliferative activity. After 24 h incubation, most of the compounds did not have any effect on cell viability, while after 72 h incubation with the compounds a decrease in cell viability was observed, but mostly at the highest tested concentrations only (>33 µM). Nevertheless, the most potent structures in this assay were **9** and **13**, the compounds that did not affect TNFα production. Finally, the antimicrobial activity of styryl-thiophene benzylamines (**2**–**8**) was examined since the iron chelating activity was confirmed for our previously synthesized heterostilbene compounds [40], and there are several references to iron chelating compounds having antibacterial properties [41,42,43]. Five different bacteria strains were treated with *cis*- and *trans-*thienostilbenes **2**–**8** in three different growth media combinations, and the minimum inhibitory concentrations (MIC) were determined. Apart from the standard antibiotics, none of the tested amine compounds had any effect on bacterial growth, regardless of bacteria strain or growth media.

## 3. Materials and Methods

### 3.1. General Remarks

All used solvents were commercially available and were purified by distillation. Anhydrous magnesium sulfate, MgSO_4,_ was used for drying organic layers after extractions. Column chromatography was performed on columns with silica gel (60 Å, technical grade). Thin-layer chromatography was performed using plates coated with silica gel (0.2 mm, Kiselgel 60 F_254_). Nuclear magnetic resonance (NMR) spectroscopic data for ^1^H and ^13^C nuclei were recorded at room temperature on Bruker Avance 300 MHz and 600 MHz spectrometers. Deuterated chloroform, CDCl_3,_ with tetramethylsilane as standard, was used for recording NMR spectra. Chemical shifts were reported in parts per million. The abbreviations used in this experimental procedure were NMR—nuclear magnetic resonance, UV—ultraviolet spectrophotometry, PE—petroleum ether, E—diethylether, ACN—acetonitrile, and EtOH—ethanol. UV spectra were recorded by UV/Vis spectrophotometer. Spectral changes during the irradiation were recorded in HPLC ACN. Before these measurements, the reaction mixture was purged with nitrogen for 15 min. Preparative photochemical reactions were performed in a closed vessel in two photochemical reactors, Rayonet and Luzchem, equipped with UV lamps of 350 nm. IR spectra were acquired using a Bruker Vertex 70 Fourier transform infrared spectrometer set on attenuated total reflectance (ATR) mode. The samples were pressed on a diamond, and the absorbance data were collected between 400 and 5000 cm^−1^ with a spectral resolution of 1 cm^−1^ and an average of 32 scans. HRMS analyses were carried out on a mass spectrometer (MALDI TOF/TOF analyzer) equipped with an Nd:YAG laser operating at 355 nm with a fitting rate of 200 Hz in the positive (H+) or negative (-H) ion reflector mode. All solvents were removed from the solutions by rotary evaporator under reduced pressure. 

### 3.2. General Procedure for the Synthesis of Starting 2-(4-Chlorostyryl)Thiophene (1)

Starting compound **1** as mixtures of *cis*- and *trans*-isomers was synthesized by the Wittig reaction. The reaction solution was purged with nitrogen for 15 min before adding the reagents. In a three-necked round-bottom flask (100 mL), a solution of the corresponding phosphonium salt (11 mmol) was dissolved in 50 mL of absolute EtOH (dried on 4 Å sieves) in a three-necked flask. The solution of sodium ethoxide (11 mmol, 1.1 eq of Na dissolved in 10 mL of absolute ethanol) was added in strictly anhydrous conditions under nitrogen dropwise. Thiophene-2-carbaldehyde (11 mmol) was added directly to a stirred solution. The reaction mixture was left to stir for 24 h at room temperature with a nitrogen balloon. After removing the solvent, absolute ethanol, by rotary evaporator under reduced pressure, the solid reaction mixture was extracted with toluene p.a. (3 × 25 mL). 

The organic layer was dried under anhydrous MgSO_4_. The product was isolated by column chromatography on silica gel using PE as eluent and characterized by spectroscopic methods. The first fractions yielded *cis*- and the last fractions *trans*-isomer.

**2-(4-chlorostyryl)thiophene (1) [34]**: column chromatography on silica gel using PE as eluent afforded 840 mg (86%) of the mixture of isomers of **1** (*cis-***1**:*trans-***1** = 2:1). Repeated column chromatography on silica gel using PE as eluent afforded pure *cis*-**1** in the first fractions and *trans*-**1** in the last fractions.


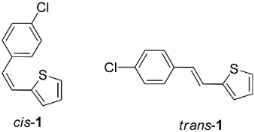


**(*Z*)-2-(4-chlorostyryl)thiophene (*cis*-1):** 470 mg (isolated, 56%), colorless oil; R*_f_*(PE) = 0.89; UV (ACN) *λ_max_*/nm (*ε*/dm^3^mol^−1^cm^−1^) 294 (9618). ^1^H NMR (CDCl_3_, 300 MHz) *δ*/ppm: 7.36–7.19 (m, 4H), 7.12 (dd, *J* = 5.1, 1.3 Hz, 1H), 7.01–6.84 (m, 2H), 6.71 (d, *J* = 12.0 Hz, 1H), 6.49 (d, *J* = 12.0 Hz, 1H). ^13^C NMR (CDCl_3_, 75 MHz) *δ*/ppm: 139.3 (s), 135.7 (s), 133.3 (s), 130.2 (d), 128.7 (d), 128.4 (d), 127.5 (d), 126.6 (d), 125.7 (d), 123.9 (d). MS (ESI) *m*/*z* (%, fragment): 221 (100).

**(*E*)-2-(4-chlorostyryl)thiophene (*trans*-1)**: 370 mg (isolated, 44%), white powder, m.p. 98–100 °C; R*_f_*(PE) = 0.85; UV (ACN) *λ_max_*/nm (*ε*/dm^3^mol^−1^cm^−1^) 327 (35,185). ^1^H NMR (CDCl_3_, 600 MHz) *δ*/ppm: 7.41–7.36 (m, 2H), 7.33–7.28 (m, 2H), 7.23–7.17 (m, 2H), 7.07 (dt, *J* = 3.4, 0.8 Hz, 1H), 7.01 (dd, *J* = 5.1, 3.6 Hz, 1H), 6.87 (d, *J* = 16.1 Hz, 1H). ^13^C NMR (CDCl_3_, 150 MHz) *δ*/ppm: 142.5 (s), 135.5 (s), 133.1 (s), 128.9 (d), 127.7 (d), 127.4 (d), 126.9 (d), 126.4 (d), 124.7 (d), 122.4 (d). MS (ESI) *m*/*z* (%, fragment): 221 (100). 

IR *v*_max_/cm^−1^ (NaCl, obtained for the pure mixture of geometrical isomers): 2910.8, 2850.7, 1726.3, 1616.7, 1488.7, 1399.6, 1082.1, 947.3. HRMS (*m*/*z*) for C_12_H_9_ClS (obtained for the pure mixture of geometrical isomers): [M+H]^+^_calcd_ = 220.0113, [M+H]^+^_measured_ = 220.0115.

### 3.3. General Procedure for the Synthesis of New 4-(2-(Thiophen-2-yl)Vinyl)Anilines (2–8) by Buchwald-Hartwig Amination

4-(2-(thiophen-2-yl)vinyl)anilines (**2**–**8**) were synthesized using **1** (0.36 mmol, 1 eq), XPhos (0.72 mmol, 0.2 eq), Pd(OAc)_2_ (0.018 mmol, 0.05 eq), and Cs_2_CO_3_ (0.54 mmol, 1.5 eq) dissolved in 2 mL of dioxane, and benzyl-amines (0.72 mmol, 2 eq) were added. The reaction mixture was purged with argon and heated to 120 °C in a pressure tube for 24 h. The reactions were performed always starting from pure isomers of **1** (except for **4** and **5**). In the case of pure *cis*-isomers, the isomerization is noticed and afforded 40–50% of the corresponding *trans*-isomers (in the case of **2**, **3,** and **7**) or 10% of *trans*-**6** and *trans*-**8**. During the amination reactions of the pure *trans*-isomers, only 10–20% of all corresponding *cis*-isomers are formed in all cases. Buchwald–Hartwig aminations with the chloro-benzyl amines were performed with the mixture of isomers of **1** (*cis*-:*trans-* = 1:1), giving the mixtures of isomers of amines **4** and **5** with the same ratios. The solvent was evaporated under pressure. The target isomer was purified by column chromatography on silica gel using PE/DCM (0–50%) as eluent afforded in the first fractions traces of **1** and the last fractions pure *cis*- and *trans*-isomers of **2**–**8**. In the case of the *meta*-methoxy derivative, the *R*_f_ values were very similar, and it was impossible to separate the pure isomers of amine **7**. 


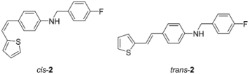


**(*Z*)-*N*-(4-fluorobenzyl)-4-(2-(thiophen-2-yl)vinyl)aniline (*cis*-2)**: 30 mg (isolated, 45%), yellow oil; R*_f_*(PE/DCM = 30%) = 0.35. UV (ACN) *λ_max_*/nm (*ε*/dm^3^mol^−1^cm^−1^) 322 (18649). ^1^H NMR (CDCl_3_, 600 MHz) *δ*/ppm: 7.36–7.31 (m, 2H), 7.23–7.18 (m, 2H), 7.09 (dd, *J* = 5.1, 1.2 Hz, 1H), 7.06–6.97 (m, 2H), 6.89 (dd, *J* = 5.1, *J* = 3.5 Hz, 1H), 6.61–6.55 (m, 1H), 6.59–6.56 (m, 2H), 6.54 (dd, *J* = 11.9, *J* = 0.8 Hz, 1H), 6.46 (d, *J* = 11.9 Hz, 1H), 4.31 (s, 2H), 4.13 (s, 1H); ^13^C NMR (CDCl_3_, 75 MHz) *δ*/ppm: 145.9 (s), 141.5 (d, *J*_C-F_ = 250 Hz), 140.2 (s), 133.5 (s), 128.9 (d), 128.4 (d), 127.4 (d), 126.5 (d), 126.1 (s), 125.4 (d), 124.4 (d), 123.8 (d), 120.1 (d), 112.0 (d), 46.7 (t). MS (ESI) *m*/*z* (%, fragment): 309 (100). 

**(*E*)-*N*-(4-fluorobenzyl)-4-(2-(thiophen-2-yl)vinyl)aniline (*trans*-2)**: 13 mg (isolated, 16%), white powder, m.p. 119–120 °C; R*_f_*(PE/DCM = 30%) = 0.32. UV (ACN) *λ_max_*/nm (*ε*/dm^3^mol^−1^cm^−1^) 355 (26710). ^1^H NMR (CDCl_3_, 600 MHz) *δ*/ppm: 7.34–7.31 (m, 2H), 7.29 (d, *J* = 8.7 Hz, 2H), 7.12 (d, *J* = 5.2 Hz, 1H), 7.04 (d, *J* = 3.2 Hz, 1H), 7.03–7.01 (m, 2H), 6.98–6.96 (m, 2H), 6.84 (d, *J* = 16.1 Hz, 1H), 6.61 (d, *J* = 8.1 Hz, 2H), 4.32 (s, 2H); ^13^C NMR (CDCl_3_, 150 MHz) *δ*/ppm: 162.0 (d, *J*_C-F_ = 245 Hz), 143.6 (s), 129.2 (d), 129.0 (d), 128.5 (d), 127.6 (d), 127.4 (d), 126.6 (s), 124.8 (d), 123.3 (d), 115.6 (d), 115.4 (d), 29.6 (t). MS (ESI) *m*/*z* (%, fragment): 309 (100). 

IR *v*_max_/cm^−1^ (NaCl, obtained for the pure mixture of geometrical isomers): 2917.7, 2850.3, 1729.8, 1600.7, 1511.6, 1223.7, 941.6. HRMS (*m*/*z*) for C_19_H_16_FNS (obtained for the pure mixture of geometrical isomers): [M+H]^+^_calcd_ = 309.0987, [M+H]^+^_measured_ = 309.0986.


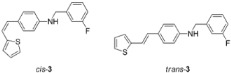


**(*Z*)-*N*-(3-fluorobenzyl)-4-(2-(thiophen-2-yl)vinyl)aniline (*cis*-3)**: 42 mg (isolated, 65%), yellow oil; R*_f_*(PE/DCM = 30%) = 0.55. UV (ACN) *λ_max_*/nm (*ε*/dm^3^mol^−1^cm^−1^) 327 (18788). ^1^H NMR (CDCl_3_, 600 MHz) *δ*/ppm: 7.32–7.28 (m, 1H), 7.19 (d, *J* = 8.3 Hz, 2H), 7.14 (d, *J* = 7.4 Hz, 1H), 7.09–7.07 (m, 2H), 6.99 (d, *J* = 4.0 Hz, 1H), 6.95 (dt, *J* = 8.6; 1.8 Hz, 1H), 6.89 (dd, *J* = 4.9; 4.0 Hz, 1H), 6.57 (d, *J* = 8.0 Hz, 2H), 6.53 (d, *J* = 12.0 Hz, 1H), 6.46 (d, *J* = 12.0 Hz, 1H), 4.36 (s, 2H), 4.16 (s, 1H); ^13^C NMR (CDCl_3_, 150 MHz) *δ*/ppm: 144.3 (d, *J*_C-F_ = 243 Hz), 140.3 (s), 139.3 (s), 133.1 (s), 130.1 (d), 129.4 (d), 128.8 (d), 128.5 (d), 127.6 (d), 126.5 (d), 125.9 (d, *J*_C-F_ = 22 Hz), 124.9 (d), 123.3 (d), 121.2 (d), 118.9 (d), 113.2 (d), 46.7 (t). MS (ESI) *m*/*z* (%, fragment): 309 (100). 

**(*E*)-*N*-(3-fluorobenzyl)-4-(2-(thiophen-2-yl)vinyl)aniline (*trans*-3)**: 61 mg (isolated, 62%), yellow powder, m.p. 95–99 °C; R*_f_* (PE/DCM = 30%) = 0.53. UV (ACN) *λ_max_*/nm (*ε*/dm^3^mol^−1^cm^−1^) 355 (38641). ^1^H NMR (CDCl_3_, 600 MHz) *δ*/ppm: 7.31–7.28 (m, 3H), 7.14 (d, *J* = 7.5 Hz, 1H), 7.11 (d, *J* = 4.7 Hz, 1H), 7.07 (d, *J* = 9.5 Hz, 1H), 7.02 (d, *J* = 16.7 Hz, 1H), 6.97–6.95 (m, 3H), 6.83 (d, *J* = 16.2 Hz, 1H), 6.58 (d, *J* = 8.6 Hz, 2H), 4.36 (s, 2H), 4.23 (s, 1H). ^13^C NMR (CDCl_3_, 150 MHz) *δ*/ppm: 163.4 (d, *J*_C-F_ = 248 Hz), 145.8 (s), 143.5 (d), 140.5 (s), 130.3 (d), 130.2 (d), 128.3 (d), 127.6 (d), 125.0 (d), 123.4 (d), 123.2 (d), 121.8 (s), 118.7 (d), 114.7 (d), 114.3 (d), 48.5 (t). MS (ESI) *m*/*z* (%, fragment): 309 (100). 

IR *v*_max_/cm^−1^ (NaCl, obtained for the pure mixture of geometrical isomers): 3375.7, 3010.2, 1598.4, 1519.6, 1463.6, 1312.8, 1253.4, 961.0, 821.5. HRMS (*m*/*z*) for C_19_H_16_FNS (obtained for the pure mixture of geometrical isomers): [M+H]^+^_calcd_ = 309.0987, [M+H]^+^_measured_ = 0985.


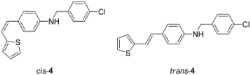


**(*Z*)-*N*-(4-chlorobenzyl)-4-(2-(thiophen-2-yl)vinyl)aniline (*cis*-4)**: 11 mg (isolated, 24%), yellow oil; R*_f_*(PE/DCM = 50%) = 0.52. UV (ACN) *λ_max_*/nm (*ε*/dm^3^mol^−1^cm^−1^) 323 (20,618), 259 (22,985). ^1^H NMR (CDCl_3_, 600 MHz) *δ*/ppm: 7.31–7.29 (m, 4H), 7.22–7.17 (m, 2H), 7.09 (dd, *J* = 5.1, 1.2 Hz, 1H), 6.99 (dt, *J* = 3.6, 1.0 Hz, 1H), 6.89 (dd, *J* = 5.1, 3.5 Hz, 1H), 6.59–6.51 (m, 3H), 6.46 (d, *J* = 11.9 Hz, 1H), 4.32 (s, 2H), 4.12 (s, 1H). ^13^C NMR (CDCl_3_, 75 MHz) *δ*/ppm: 147.0 (s), 144.0 (s), 140.4 (s), 137.7 (s), 133.0 (s), 130.1 (d), 129.5 (d), 128.8 (d), 128.6 (d), 127.5 (d), 126.5 (d), 124.8 (d), 121.2 (d), 112.8 (d), 47.9 (t). MS (ESI) *m*/*z* (%, fragment): 326 (100). 

**(*E*)-*N*-(4-chlorobenzyl)-4-(2-(thiophen-2-yl)vinyl)aniline (*trans*-4)**: 22 mg (isolated, 51%), white powder, m.p. 128–130 °C; R*_f_*(PE/DCM = 50%) = 0.47. UV (ACN) *λ_max_*/nm (*ε*/dm^3^mol^−1^cm^−1^) 357 (27,719), 338 (sh, 14,015). ^1^H NMR (CDCl_3_, 600 MHz) *δ*/ppm: 7.32–7.27 (m, 6H), 7.13 (d, *J* = 4.5 Hz, 1H), 7.05 (d, *J* = 16.2 Hz, 1H), 6.98–6.95 (m, 2H), 6.83 (d, *J* = 16.3 Hz, 1H), 6.69 (d, *J* = 8.1 Hz, 2H), 4.34 (s, 2H). ^13^C NMR (CDCl_3_, 150 MHz) *δ*/ppm: 143.6 (s), 137.3 (s), 133.2 (s), 130.1 (s), 128.9 (d), 128.8 (d), 128.7 (s), 128.4 (d), 127.6 (d), 127.5 (d), 124.9 (d), 123.3 (d), 118.4 (d), 113.6 (d), 47.9 (t). MS (ESI) *m*/*z* (%, fragment): 326 (100). 

HRMS (*m*/*z*) for C_19_H_16_ClNS (obtained for the pure mixture of geometrical isomers): [M+H]^+^_calcd_ = 325.0692, [M+H]^+^_measured_ = 325.0685.


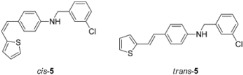


**(*Z*)-*N*-(3-chlorobenzyl)-4-(2-(thiophen-2-yl)vinyl)aniline (*cis*-5)**: 13 mg (isolated, 36%), yellow oil; R*_f_*(PE/DCM = 50%) = 0.50. UV (ACN) *λ_max_*/nm (*ε*/dm^3^mol^−1^cm^−1^) 325 (15,472), 259 (18,329). ^1^H NMR (CDCl_3_, 600 MHz) *δ*/ppm: 7.23–7.15 (m, 4H), 7.15–7.10 (m, 2H), 7.02 (dd, *J* = 5.1, 1.2 Hz, 1H), 6.92 (dt, *J* = 3.6, 1.0 Hz, 1H), 6.82 (dd, *J* = 5.1, 3.6 Hz, 1H), 6.52–6.47 (m, 2H), 6.47 (d, *J* = 11.9, 1H), 6.39 (d, *J* = 11.9 Hz, 1H), 4.26 (s, 2H), 4.08 (s, 1H). ^13^C NMR (CDCl_3_, 75 MHz) *δ*/ppm: 146.1 (s), 145.8 (s), 142.7 (s), 140.2 (s), 139.3 (s), 133.5 (s), 128.9 (d), 128.3 (d), 127.5 (d), 126.5 (d), 126.1 (s), 125.4 (d), 124.5 (d), 123.8 (d), 122.2 (d), 120.3 (d), 117.2 (d), 112.0 (d), 46.7 (t). MS (ESI) *m*/*z* (%, fragment): 326 (100). 

**(*E*)-*N*-(3-chlorobenzyl)-4-(2-(thiophen-2-yl)vinyl)aniline (*trans*-5)**: 15 mg (isolated, 41%), yellow powder, m.p. 123–125 °C; R*_f_*(PE/DCM = 50%) = 0.46. UV (ACN) *λ_max_*/nm (*ε*/dm^3^mol^−1^cm^−1^) 355 (37968). ^1^H NMR (CDCl_3_, 600 MHz) *δ*/ppm: 7.25–7.20 (m, 2H), 7.20–7.15 (m, 4H), 7.06–7.02 (m, 1H), 6.95 (dd, *J* = 16.1, 0.8 Hz, 1H), 6.92–6.87 (m, 2H), 6.76 (d, *J* = 16.1 Hz, 1H), 6.54–6.48 (m, 2H), 4.27 (s, 2H), 4.14 (s, 1H). ^13^C NMR (CDCl_3_, 75 MHz) *δ*/ppm: 146.2 (s), 142.7 (s), 140.2 (s), 139.4 (s), 133.5 (s), 128.9 (d), 128.4 (s), 127.5 (d), 126.5 (d), 126.3 (d), 126.0 (d), 125.4 (d), 124.4 (d), 123.8 (d), 122.2 (d), 120.2 (s), 117.1 (d), 111.9 (d), 46.7 (t). MS (ESI) *m*/*z* (%, fragment): 326 (100). 

HRMS (*m*/*z*) for C_19_H_16_ClNS (obtained for the pure mixture of geometrical isomers): [M+H]^+^_calcd_ = 325.0692, [M+H]^+^_measured_ = 325.0686.


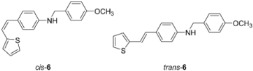


**(*Z*)-*N*-(4-methoxybenzyl)-4-(2-(thiophen-2-yl)vinyl)aniline (*cis*-6)**: 95 mg (isolated, 53%), yellow oil; R*_f_*(PE/DCM = 50%) = 0.43. UV (ACN) *λ_max_*/nm (*ε*/dm^3^mol^−1^cm^−1^) 325 (17,223). ^1^H NMR (CDCl_3_, 600 MHz) *δ*/ppm: 7.26 (d, *J* = 8.8 Hz, 2H), 7.18 (d, *J* = 8.4 Hz, 2H), 7.06 (dd, *J* = 5.1, 1.2 Hz, 1H), 6.98 (dt, *J* = 3.6, 1.0 Hz, 1H), 6.89–6.84 (m, 3H), 6.55 (d, *J* = 8.6 Hz, 2H), 6.51 (dd, *J* = 11.9, 0.8 Hz, 1H), 6.44 (d, *J* = 11.9 Hz, 1H), 4.23 (s, 2H), 4.98 (s, 1H), 3.77 (s, 3H). ^13^C NMR (CDCl_3_, 75 MHz) *δ*/ppm: 158.9 (s), 147.7 (s), 140.6 (s), 131.2 (s), 130.1 (d), 129.7 (d), 128.9 (d), 127.6 (d), 126.5 (d), 126.2 (s), 124.8 (d), 120.9 (d), 114.1 (d), 112.6 (d), 55.3 (t), 47.8 (q). MS (ESI) *m*/*z* (%, fragment): 321 (100).

**(*E*)-*N*-(4-methoxybenzyl)-4-(2-(thiophen-2-yl)vinyl)aniline (*trans*-6)**: 11 mg (isolated, 12%), yellow powder, m.p. 99–102 °C; R*_f_*(PE/DCM = 50%) = 0.37. UV (ACN) *λ_max_*/nm (*ε*/dm^3^mol^−1^cm^−1^) 358 (38,016). ^1^H NMR (CDCl_3_, 600 MHz) *δ*/ppm: 7.32–7.26 (m, 4H), 7.11 (dd, *J* = 4.7, 1.1 Hz, 1H), 7.05 (d, *J* = 16.4 Hz, 1H), 6.99–6.94 (m, 2H), 6.91–6.87 (m, 2H), 6.84 (d, *J* = 16.4 Hz, 1H), 6.63–6.58 (m, 2H), 4.27 (s, 2H), 3.80 (s, 3H). ^13^C NMR (CDCl_3_, 150 MHz) *δ*/ppm: 159.2 (s), 143.6 (s), 134.0 (s), 130.1 (s), 129.6 (d), 129.4 (d), 128.4 (d), 127.5 (d), 126.5 (s), 125.1 (d), 123.4 (d), 118.7 (d), 114.7 (d), 114.1 (d), 55.3 (t), 48.9 (q). MS (ESI) *m*/*z* (%, fragment): 321 (100). 

IR *v*_max_/cm^−1^ (NaCl, obtained for the pure mixture of geometrical isomers): 3414.5, 2994.2, 2934.3, 1600.7, 1508.2, 1332.2, 1232.9, 1178.1, 1034.7, 813.7. HRMS (*m*/*z*) for C_20_H_19_NOS (obtained for the pure mixture of geometrical isomers): [M+H]^+^_calcd_ = 321.1187, [M+H]^+^_measured_ = 321.1190.


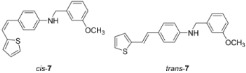


**(*Z*)-*N*-(3-methoxybenzyl)-4-(2-(thiophen-2-yl)vinyl)aniline (*cis*-7):** R*_f_*(PE/DCM= 50%) = 0.50. **(*E*)-*N*-(3-methoxybenzyl)-4-(2-(thiophen-2-yl)vinyl)aniline (*trans*-7):** R*_f_*(PE/DCM = 50%) = 0.46. 

IR *v*_max_/cm^−1^ (NaCl, obtained for the pure mixture of geometrical isomers): 3401.9, 2839.9, 1710.3, 1601.8, 1516.2, 1463.6, 1321.9, 1256.9, 1148.4, 1046.7, 777.1. HRMS (*m*/*z*) for C_20_H_19_NOS (obtained for the pure mixture of geometrical isomers): [M+H]^+^_calcd_ = 321.1187, [M+H]^+^_measured_ = 321.1185.


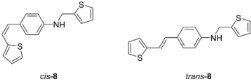


**(*Z*)-4-(2-(thiophen-2-yl)vinyl)-*N*-(thiophen-2-ylmethyl)aniline (*cis*-8)**: 72.5 mg (isolated, 74%), yellow oil; R*_f_*(PE/DCM(50%)) = 0.60. UV (ACN) *λ_max_*/nm (*ε*/dm^3^mol^−1^cm^−1^) 325 (14,931). IR *v*_max_/cm^−1^ (NaCl): 3395.5, 3017.0, 2852.6, 1594.9, 1519.6, 1393.9, 1318.6, 1250.0, 1164.3, 822.8. ^1^H NMR (CDCl_3_, 300 MHz) *δ*/ppm: 7.20 (d, *J* = 7.8 Hz, 3H), 7.07 (dd, *J* = 5.1, 1.2 Hz, 1H), 7.00–6.93 (m, 3H), 6.88 (dd, *J* = 5.1, 3.6 Hz, 1H), 6.61 (d, *J* = 8.8 Hz, 2H), 6.54 (d, *J* = 11.9 Hz, 1H), 6.45 (d, *J* = 11.9 Hz, 1H), 4.50 (s, 2H), 4.09 (s, 1H). ^13^C NMR (CDCl_3_, 75 MHz) *δ*/ppm: 147.1 (s), 142.6 (s), 140.5 (s), 130.1 (d), 129.5 (d), 127.5 (d), 126.9 (d), 126.8 (s), 126.5 (d), 125.2 (d), 124.8 (d), 124.7 (d), 121.2 (d), 112.9 (d), 43.4 (t). MS (ESI) *m*/*z* (%, fragment): 297 (100). 

**(*E*)-4-(2-(thiophen-2-yl)vinyl)-*N*-(thiophen-2-ylmethyl)aniline (*trans*-8)**: 74 mg (isolated, 64%), yellow powder, m.p. 103–105 °C; R*_f_*(PE/DCM(50%)) = 0.58. UV (ACN) *λ_max_*/nm (*ε*/dm^3^mol^−1^cm^−1^) 355 (34,147). ^1^H NMR (CDCl_3_, 600 MHz) *δ*/ppm: 7.29 (d, *J* = 8.3 Hz, 2H), 7.19 (dd, *J* = 5.6, 1.9 Hz, 1H), 7.09 (dd, *J* = 5.5, 1.1 Hz 1H), 7.01 (d, *J* = 16.1 Hz, 1H), 6.99–6.98 (m, 1H), 6.96–6.94 (m, 3H), 6.83 (d, *J* = 16.1 Hz, 1H), 6.61 (d, *J* = 8.4 Hz, 2H), 4.50 (d, *J* = 1.0 Hz, 2H), 4.14 (s, 1H). ^13^C NMR (CDCl_3_, 75 MHz) *δ*/ppm: 147.2 (s), 143.7 (s), 142.6 (s), 128.6 (d), 127.7 (d), 127.6 (d), 127.5 (d), 127.1 (s), 126.9 (d), 124.8 (d), 124.7 (d), 123.2 (d), 118.1 (d), 113.2 (d), 43.4 (t). MS (ESI) *m*/*z* (%, fragment): 297 (100).

HRMS (*m*/*z*) for C_17_H_15_NS_2_ (obtained for the pure mixture of geometrical isomers): [M+H]^+^_calcd_ = 297.0645, [M+H]^+^_measured_ = 297.0646.

### 3.4. General Procedure for the Synthesis of the Electrocyclization Photoproducts **9**–**15**

A mixture of isomers of compounds **1**–**8** was dissolved in toluene p.a. (~2.5 × 10^−3^ M) and transferred to a quartz vessel (50 mL) with the addition of a catalytic amount of iodine and irradiated with 10 UV lamps at 365 nm in a Rayonet photochemical reactor for 2–4 h to achieve almost complete conversion. After removing the solvent by a rotary evaporator under reduced pressure, the photoproducts **9**–**15** were purified by column chromatography using PE/DCM (40%) as eluent from the traces of the starting substrates (in the first fractions) and completely spectroscopically characterized by NMR, UV, and HRMS measurements.


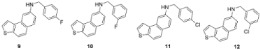


***N*-(4-fluorobenzyl)naphtho [2,1-*b*]thiophen-8-amine (9):** 12.7 mg (isolated, 69%), brown oil; R*_f_*(PE/DCM(50%)) = 0.35. UV (ACN) *λ_max_*/nm (*ε*/dm^3^mol^−1^cm^−1^) 313 (13012), 302 (10981); ^1^H NMR (CDCl_3_, 300 MHz) *δ*/ppm: 7.77 (d, *J* = 4.9 Hz, 1H), 7.72 (d, *J* = 9.1 Hz, 1H), 7.62 (d, *J* = 8.7 Hz, 1H), 7.57 (d, *J* = 8.6 Hz, 1H), 7.47 (d, *J* = 5.4 Hz, 1H), 7.40 (dd, *J* = 8.8, 5.8 Hz, 2H), 7.32 (d, *J* = 2.4 Hz, 1H), 7.04 (t, *J* = 8.7 Hz, 2H), 6.93 (dd, *J* = 8.7, 2.4 Hz, 1H), 4.70 (bs, 1H), 4.46 (s, 2H). ^13^C NMR (CDCl_3_, 150 MHz) *δ*/ppm: 162.2 (d, *J*_C-F_ = 241 Hz), 145.0 (s), 141.3 (s), 138.1 (d), 134.6 (d), 130.7 (s), 129.7 (d), 129.5 (d), 129.6 (d), 124.9 (d), 124.8 (d), 124.8 (d), 121.9 (d), 117.2 (s), 116.1 (s), 115.7 (d), 115.5 (d), 48.4 (t). MS (ESI) *m*/*z* (%, fragment): 307 (100). HRMS (*m*/*z*) for C_19_H_14_NOS: [M+H]^+^_calcd_ = 307.0831, [M+H]^+^_measured_ = 307.0833.

***N*-(3-fluorobenzyl)naphtho [2,1-*b*]thiophen-8-amine (10)**: 17.4 mg (isolated, 76%), brown oil; R*_f_*(PE/DCM(30%)) = 0.53. UV (ACN) *λ_max_*/nm (*ε*/dm^3^mol^−1^cm^−1^) 312 (13455), 303 (11232). ^1^H NMR (CDCl_3_, 300 MHz) *δ*/ppm: 7.76–7.70 (m, 2H), 7.62 (d, *J* = 8.7 Hz, 1H), 7.57 (d, *J* = 8.7 Hz, 1H), 7.46 (d, *J* = 5.4 Hz, 1H), 7.36–7.27 (m, 2H), 7.24–7.19 (m, 1H), 7.14 (d, *J* = 9.3 Hz, 1H), 6.98 (d, *J* = 7.9 Hz, 1H), 6.92 (d, *J* = 9.3 Hz, 1H), 4.53 (bs, 1H), 4.49 (s, 2H). ^13^C NMR (CDCl_3_, 150 MHz) *δ*/ppm: 163.1 (d, *J*_C-F_ = 244 Hz), 145.3 (s), 141.3 (s), 138.6 (d), 134.6 (d), 130.7 (d), 130.3 (d), 129.8 (d), 124.9 (d), 124.8 (s), 123.1 (d), 122.1 (d), 117.2 (d), 116.0 (d), 114.6 (d), 114.3 (d), 103.3 (s), 48.4 (t). MS (ESI) *m*/*z* (%, fragment): 307 (100). HRMS (*m*/*z*) for C_19_H_14_FNS: [M+H]^+^_calcd_ = 307.0831, [M+H]^+^_measured_ = 307.0834.

***N*-(4-chlorobenzyl)naphtho [2,1-*b*]thiophen-8-amine (11)**: 72.5 mg (isolated, 74%), brown oil; R*_f_*(PE/DCM = 50%) = 0.60. UV (ACN) *λ_max_*/nm (*ε*/dm^3^mol^−1^cm^−1^) 256 (13795), 245 (15007). ^1^H NMR (CDCl_3_, 300 MHz) *δ*/ppm: 7.77–7.72 (m, 2H), 7.63 (d, *J* = 8.8 Hz, 1H), 7.58 (d, *J* = 8.5 Hz, 1H), 7.47 (d, *J* = 5.7 Hz, 1H), 7.40–7.29 (m, 5H), 6.92 (dd, *J* = 8.7; 2.3 Hz, 1H), 4.50 (s, 2H), 4.38 (bs, 1H). ^13^C NMR (CDCl_3_, 75 MHz) *δ*/ppm: 147.1 (s), 142.6 (s), 140.5 (s), 130.7 (d), 130.1 (d), 129.5 (d), 127.5 (s), 126.9 (d), 126.8 (s), 126.5 (d), 125.2 (d), 124.9 (d), 124.8 (d), 124.7 (d), 121.2 (d), 112.9 (d), 43.4 (t). MS (ESI) *m*/*z* (%, fragment): 324 (100). HRMS (*m*/*z*) for C_19_H_14_ClNS: [M+H]^+^_calcd_ = 323.0535, [M+H]^+^_measured_ = 323.0526.

***N*-(3-chlorobenzyl)naphtho [2,1-*b*]thiophen-8-amine (12)**: 62.0 mg (isolated, 65%), yellow oil; R*_f_* (PE/DCM = 50%) = 0.60. UV (ACN) *λ_max_*/nm (*ε*/dm^3^mol^−1^cm^−1^) 258 (19233), 246 (21655). ^1^H NMR (CDCl_3_, 300 MHz) *δ*/ppm: 7.78–7.72 (m, 2H), 7.64 (d, *J* = 8.5 Hz, 1H), 7.58 (d, *J* = 9.0 Hz, 1H), 7.49–7.45 (m, 2H), 7.34–7.26 (m, 5H), 6.95 (dd, *J* = 9.1; 2.8 Hz, 1H), 4.81 (bs, 1H), 4.50 (s, 2H). ^13^C NMR (CDCl_3_, 150 MHz) *δ*/ppm: 147.1 (s), 142.6 (s), 140.5 (s), 130.1 (d), 129.5 (d), 127.5 (d), 126.9 (d), 126.8 (s), 126.5 (d), 125.2 (d), 124.8 (d), 124.7 (d), 121.2 (d), 112.9 (d), 43.4 (t). MS (ESI) *m*/*z* (%, fragment): 324 (100). HRMS (*m*/*z*) for C_19_H_14_ClNS: [M+H]^+^_calcd_ = 323.0535, [M+H]^+^_measured_ = 323.0544.


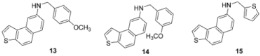


***N*-(4-methoxybenzyl)naphtho [2,1-*b*]thiophen-8-amine (13)**: 6.4 mg (isolated, 12%), brown oil; R*_f_*(PE/DCM = 50%) = 0.37. UV (ACN) *λ_max_*/nm (*ε*/dm^3^mol^−1^cm^−1^) 315 (10554), 259 (15515), 244 (12209). ^1^H NMR (CDCl_3_, 600 MHz) *δ*/ppm: 7.84 (d, *J* = 8.8 Hz, 1H), 7.81 (d, *J* = 5.6, Hz, 1H), 7.72 (d, *J* = 8.7 Hz, 1H), 7.62 (d, *J* = 8.6 Hz, 1H), 7.58 (d, *J* = 9.1 Hz, 1H), 7.48 (d, *J* = 5.4 Hz, 1H), 7.38–7.35 (m, 3H), 7.01 (d, *J* = 9.0 Hz, 1H), 6.94 (dd, *J* = 8.7; 2.3 Hz, 1H), 4.43 (s, 2H), 3.81 (s, 3H). MS (ESI) *m*/*z* (%, fragment): 319 (100). 

***N*-(3-methoxybenzyl)naphtho [2,1-*b*]thiophen-8-amine (14)**: 8.1 mg (isolated, 65%), brown oil; R*_f_*(PE/DCM = 50%) = 0.46. UV (ACN) *λ_max_*/nm (*ε*/dm^3^mol^−1^cm^−1^) 314 (12155), 279 (13610). ^1^H NMR (CDCl_3_, 300 MHz) *δ*/ppm: 8.02–7.88 (m, 3H), 7.71–7.64 (m, 2H), 7.55–7.52 (m, 1H), 7.49–7.37 (m, 5H), 5.02 (s, 2H), 3.97 (s, 1H), 3.87 (s, 3H). MS (ESI) *m*/*z* (%, fragment): 319 (100). 

***N*-(thiophen-2-ylmethyl)naphtho [2,1-*b*]thiophen-8-amine (15)**: 18.8 mg (isolated, 78%), brown oil; R*_f_*(PE/DCM = 50%) = 0.58. UV (ACN) *λ_max_*/nm (*ε*/dm^3^mol^−1^cm^−1^) 310 (10220), 256 (10868), 245 (16580). ^1^H NMR (CDCl_3_, 300 MHz) *δ*/ppm: 7.74 (d, *J* = 5.4 Hz, 1H), 7.65 (d, *J* = 8.7 Hz, 1H), 7.57 (d, *J* = 8.7 Hz, 1H), 7.49 (d, *J* = 8.7 Hz, 1H), 7.41–7.39 (m, 2H), 7.16–7.13 (m, 1H), 7.02 (d, *J* = 3.3 Hz, 1H), 6.92–6.88 (m, 2H), 5.48 (bs, 1H), 4.60 (s, 2H). ^13^C NMR (CDCl_3_, 150 MHz) *δ*/ppm: 138.1 (s), 136.3 (s), 135.1 (s), 135.1 (d), 129.9 (d), 129.8 (d), 128.3 (d), 127.2 (d), 126.5 (d), 125.6 (d), 124.5 (d), 122.1 (d), 119.4 (d), 118.0 (d), 46.6 (t); the other 3 singlets are not reliable due to the impurities in the spectrum. MS (ESI) *m*/*z* (%, fragment): 295 (100). HRMS (*m*/*z*) for C_17_H_13_NS_2_: [M+H]^+^_calcd_ = 295.0489, [M+H]^+^_measured_ = 295.0487.

### 3.5. Computational Details

Geometry optimizations of compounds **2**–**15** were performed using the Gaussian16 program package [44] at the M06-2X/6-31G(d) level of theory. Vibrational analysis was performed to verify the minima on the potential energy surface for all structures. UV-Vis spectra were computationally obtained using the (CPCM)TD-CAM-B3LYP/6–31++G(d)//M06-2X/6-31G(d) model. The conductor-like polarizable continuum model of solvation (CPCM) [45] was used to describe the solvent effect of acetonitrile. Molecular docking was performed using the Autodock program suite [39], utilizing the crystal structure of AChE, PDB code 1EEA [46], and human BChE, PDB code 1p0i [47]. The Lamarckian genetic algorithm was used, with 25 requested genetic algorithm dockings with 25 binding poses for each ligand. The most stable complexes between the ligand and the enzyme obtained by docking were optimized using a semiempirical PM6 model [48] combined with a continuum-cluster approach [49].

### 3.6. Cholinesterase Inhibition Activity Measurements

Inhibition of AChE and BChE was determined using a modified Ellman’s method [50]. Acetylthiocholine iodide (ATChI), S-butyrylthiocholine iodide (BTChI), acetylcholinesterase from electric eel, butyrylcholinesterase from equine serum, Tris-HCl buffer, and Galantamine were purchased from Sigma-Aldrich (St. Louis, MO, USA). Ellman’s reagent 5,50-dithiobis-(2-nitrobenzoic acid) (DTNB) was purchased from Zwijndrecht (Antwerpen, Belgium). Galantamine was used as a reference standard. A mixture of 180 µL Tris HCl buffer (50 mM, pH 8.0), 10 µL of AChE/BChE prepared in 20 mM Tris HCl buffer, pH 7.5 (final concentration 0.03 U/mL), and 10 µL of tested solution (final concentrations 20–250 μM in ethanol) was pre-incubated for 5 min at 4 °C. The reaction started with adding 10 μL of DTNB (final concentration 0.3 mM prepared in Tris buffer) and 10 μL of ATChI/BTChI (final concentration of 0.5 mM prepared in Tris buffer). The developing yellow color was measured at 405 nm over 6 min at room temperature using a 96-well microplate reader (IRE 96, SFRI Medical Diagnostics). The experiment was run in triplicate. Percentage enzyme inhibition was calculated according to the equation:

Inhibition (%) = [(*A*_C_ − *A*_T_)/*A*_C_] × 100

where *A*_C_ is the enzyme activity without the test sample and *A*_T_ is the enzyme activity with the test sample, represented as mean values ± standard deviation. Non-enzymatic hydrolysis was measured as blank for control measurement without inhibitors. The non-enzymatic hydrolysis reaction with added inhibitor was used as a blank for the samples. The IC_50_ value was calculated by a nonlinear fit of compound concentration (log) values vs. response. 

### 3.7. In Vitro Biological Activity Evaluation

**TNFα production in LPS stimulated PBMCs:** human peripheral blood mononuclear cells (PBMCs) were isolated and treated with compounds, and the effects on TNFa production evaluated as described previously [28]. 

**THP-1 cell viability assay:** THP-1 cell line (TIB-202, ATCC) was maintained in culture medium (RPMI1640 + 10% FBS). For the experiment, cells were seeded into 96-well plates, 30,000 cells/well, and treated with compounds prepared as described for PBMC assay. As reference, the compound staurosporine was used, with starting concentration set to 1 µM. Cells were incubated with compounds for 24 h, 48 h, and 72 h at 37 °C, 5% CO_2_, and 95% humidity. At each time point, cell viability was determined using CellTiter Glo assay, following the manufacturer’s instructions.

**Evaluation of antibacterial activity (MIC value determination):** standard antibiotics (ciprofloxacin and ceftazidime, both purchased from USP, USA) were prepared according to CLSI guidelines, while test compounds were prepared as 5 mg/mL DMSO solutions. Upon DMSO stock solutions preparation, the working solutions in media were prepared. Compounds were tested in three different growth media, cation-adjusted Mueller–Hinton broth (CA-MHB, Becton Dickinson, USA), Mueller–Hinton broth (Sigma, Germany), and RPMI1640 medium. From working solutions, dilutions in growth medium were prepared and the compounds and antibiotics were plated in 96-well assay plates. All compounds were tested in serial twofold dilutions, giving a final concentration range of 64–0.125 µg/mL. Microorganisms (*S. aureus* ATCC 29213, *E. coli* ATCC 25922, *K. pneumoniae* ATCC 700603, *P. aeruginosa* ATCC 27853, and *A. baumannii* ATCC 17978) were revived from glycerol stock kept at −70 °C by plating them on MH agar plates. The following day, the single colony of each microorganism was streaked on fresh agar plates. The next day, using the direct colony suspension method, broth solutions that achieved turbidity equivalent to the 0.5 McFarland standard for each microorganism were prepared. This resulted in suspensions containing 1–2 × 10^8^ CFU/mL. Out of these suspensions, actual inoculums were prepared by diluting them 100x with media, giving a final microorganism count of 2–8 × 10^5^ CFU/mL, and seeded into 96-well plates and treated with compounds. The plates were incubated for 16–24 h at 37 °C. MIC values were determined by visual inspection of bacterial growth within 96-well plates. The first column in which there is no visible growth of bacteria is determined to be the MIC value for the compound or antibiotic tested in that particular row. ATCC quality control strains are used as referent strains for which there are designated MIC values for standard antibiotics. In this way, quality control of the assay is determined. The assay is considered valid when MIC values for standard antibiotics are within the CLSI-designated range for the ATCC strain tested.

## 4. Conclusions

In this research, we synthesized a whole series of new *cis*- and *trans*-isomers of amino-stilbenes **2**–**8** to test the efficiency of their production, acid resistance, photochemical and photophysical characteristics, and the initial difference in the potential biological activity of the starting compounds **2**–**8** and their photocyclization products **9**–**15**. Given that heterostilbene amine of *cis*-configuration, *cis*-**8**, and its photoproduct with two thiophene cores, **15**, were shown to be promising ChE inhibitors, it is worth functionalizing them in further research to achieve more pronounced biological properties. As already concluded from our previous results [28], the thiophene’s key role in inhibiting enzyme cholinesterases (especially BChE) in combination with the *cis*-configuration or the planar thienonaphthalene part of the molecule is confirmed in this study, too. The comparison with organic dyes possessing an amino-stilbene subunit as the scaffold [30] shows that the new *trans*-aminostilbenes (*trans*-**2**–**8**) have a very similar absorbance wavelength. They are also insensitive to the change in pH toward lower values. In this sense, such a suitable *trans*-styryl skeleton can be the starting point in future chemical modifications for the synthesis of new aromatic amines and their transformation into ammonium salts, evaluating them as styryl dyes.

## Figures and Tables

**Table 1:** Measured and calculated values of absorption maxima in UV-Vis spectra of *cis*-styryl-thiophene benzylamines, along with transitions responsible for *λ*_max_. Calculated data were obtained at the computational model (CPCM)TD-CAM-B3LYP/6–31++G(d) (ACN), with geometries optimized at the M06-2X/6-31G(d) level of theory. The highest occupied and lowest unoccupied molecular orbital are denoted by H and L, respectively.

Compound	*λ*^exp^/nm	*λ*^calc^/nm	Assignment
*cis*-**2**	322	323	H → L
*cis*-**3**	327	323	H → L
*cis*-**4**	323	323	H → L
*cis*-**5**	325	323	H → L
*cis*-**6**	327	325	H → L
*cis*-**7**	- ^1^	324	H → L
*cis*-**8**	325	322	H → L

^1^ Isomers could not be separated.

**Table 2:** Measured and calculated *λ*_max_ values for *trans*-styryl-thiophene benzylamines, along with main assignments. Calculated data is obtained at the computational model (CPCM)TD-CAM-B3LYP/6–31++G(d) (ACN), with geometries optimized at the M06-2X/6-31G(d) level of theory. The highest occupied and lowest unoccupied molecular orbital are denoted by H and L, respectively.

Compound	*λ*^exp^/nm	*λ*^calc^/nm	Assignment
*trans*-**2**	355	340	H → L
*trans*-**3**	355	340	H → L
*trans*-**4**	357	340	H → L
*trans*-**5**	355	340	H → L
*trans*-**6**	358	342	H → L
*trans*-**7**	- ^1^	341	H → L
*trans*-**8**	355	339	H → L

^1^ Isomers could not be separated.

## Data Availability

Additional data is avalilable on request.

## Supplementary Materials

The following supporting information can be downloaded at https://www.mdpi.com/article/10.3390/ijms24010610/s1.
