# Supplementary material for: Synthesis, Photochemistry, Computational Study and Potential Application of New Styryl-Thiophene and Naphtho-Thiophene Benzylamines
Source: Int J Mol Sci. 2022 Dec 29;24(1):610. doi: 10.3390/ijms24010610 (PMC9820070; doi:10.3390/ijms24010610)

***Electronic Supporting Information***

**Synthesis, photochemistry, computational study and potential application of new styryl-thiophene and naphtho-thiophene benzylamines**

**Milena Mlakić ^1^, Ilijana Odak ^2^, Ivan Faraho ^3^, Martina Bosnar ^3^, Mihailo Banjanac ^3^, Zlata Lasić ^4^, Željko Marinić ^5^, Danijela Barić ^6,^* and Irena Škorić ^1,^***

^1^ Department of Organic Chemistry, Faculty of Chemical Engineering and Technology, University of Zagreb, Marulićev trg 19, HR-10000 Zagreb, Croatia; [mdragojev@fkit.hr](mailto:mdragojev@fkit.hr) (M.M.)

^2^ Department of Chemistry, Faculty of Science and Education, University of Mostar, Matice hrvatske bb, 88 000 Mostar, Bosnia and Herzegovina; ilijana.odak@fpmoz.sum.ba (I.O.)

^3^ Pharmacology *in vitro*, Selvita Ltd., Prilaz baruna Filipovića 29, HR-10 000 Zagreb, Croatia; [Ivan.Faraho@selvita.com](mailto:Ivan.Faraho@selvita.com) (I.F.); [Martina.Bosnar@selvita.com](mailto:Martina.Bosnar@selvita.com) (M.B.); Mihailo.Banjanac@selvita.com (M.Ba.)

^4^ Teva api Analytical R&D, Pliva, Prilaz Baruna Filipovića 25, HR-10 000 Zagreb, Croatia; Zlata.Lasic01@pliva.com (Z.L.)

^5^ NMR Center, Ruđer Bošković Institute, Bijenička cesta 54, HR-10 000 Zagreb, Croatia; [zmarinic@irb.hr](mailto:zmarinic@irb.hr) (Z.M.)

^6^ Group for Computational Life Sciences, Division of Physical Chemistry, Ruđer Bošković Institute, Bijenička cesta 54, HR-10 000 Zagreb, Croatia

***** Correspondence: dbaric@irb.hr (D.B.); iskoric@fkit.hr (I.Š.)
Tel.: +385-1-4571-385 (D.B.); +385-1-4597-241 (I.Š.)

**Content:**

1. ^1^H and ^13^C NMR spectra of synthesized compounds **2-15** (Figs S1-S62)

2. MS spectra and HRMS analyses of synthesized compounds **2-15** (Figs S63-S73)

3. IR (ATR) spectra of synthesized compounds **2-15** (Figs S74-S79)

4. Cartesian coordinates of compounds **2**-**15**

5. Cartesian coordinates of complexes of *cis*-**8** and **15** with the active site of AChE and BChE, respectively

6. Calculated UV-Vis spectra of compounds **2-15**

**1. ^1^H and ^13^C NMR spectra of synthesized compounds 2-15**

**Figure S1.** ^1^H NMR spectrum (CDCl_3_) of (*Z*)-2-(4-chlorostyryl)thiophene (*cis*-**1**).


**Figure S2.** Part of the ^1^H NMR spectrum (CDCl_3_) of (*Z*)-2-(4-chlorostyryl)thiophene (*cis*-**1**).


**Figure S3.** ^13^C NMR spectrum (CDCl_3_) of (*Z*)-2-(4-chlorostyryl)thiophene (*cis*-**1**).


**Figure S4.** ^1^H NMR spectrum (CDCl_3_) of (*E*)-2-(4-chlorostyryl)thiophene (*trans*-**1**).

 **Figure S5.** Part of the ^1^H NMR spectrum (CDCl_3_) of (*E*)-2-(4-chlorostyryl)thiophene (*trans*-**1**).

**Figure S6.** ^13^C NMR spectrum (CDCl_3_) of (*E*)-2-(4-chlorostyryl)thiophene (*trans*-**1**).

**Figure S7.** ^1^H NMR spectrum (CDCl_3_) of (*Z*)-*N*-(4-fluorobenzyl)-4-(2-(thiophen-2-yl)vinyl)aniline (*cis*-**2**).


**Figure S8.** Part of the ^1^H NMR spectrum (CDCl_3_) of (*Z*)-*N*-(4-fluorobenzyl)-4-(2-(thiophen-2-yl)vinyl)aniline (*cis*-**2**).

**Figure S9.** APT NMR spectrum (CDCl_3_) of (*Z*)-*N*-(4-fluorobenzyl)-4-(2-(thiophen-2-yl)vinyl)aniline (*cis*-**2**).


**Figure S10.** ^1^H NMR spectrum (CDCl_3_) of (*E*)-*N*-(4-fluorobenzyl)-4-(2-(thiophen-2-yl)vinyl)aniline (*trans*-**2**).

**Figure S11.** Part of the ^1^H NMR spectrum (CDCl_3_) of (*E*)-*N*-(4-fluorobenzyl)-4-(2-(thiophen-2-yl)vinyl)aniline (*trans*-**2**).

**Figure S12.** APT NMR spectrum (CDCl_3_) of (*E*)-*N*-(4-fluorobenzyl)-4-(2-(thiophen-2-yl)vinyl)aniline (*trans*-**2**).

**Figure S13.** ^1^H NMR spectrum (CDCl_3_) of (*Z*)-*N*-(3-fluorobenzyl)-4-(2-(thiophen-2-yl)vinyl)aniline (*cis*-**3**).

**Figure S14.** Part of the ^1^H NMR spectrum (CDCl_3_) of (*Z*)-*N*-(3-fluorobenzyl)-4-(2-(thiophen-2-yl)vinyl)aniline (*cis*-**3**).

**Figure S15.** APT NMR spectrum (CDCl_3_) of (*Z*)-*N*-(3-fluorobenzyl)-4-(2-(thiophen-2-yl)vinyl)aniline (*cis*-**3**).

**Figure S16.** ^1^H NMR spectrum (CDCl_3_) of (*E*)-*N*-(3-fluorobenzyl)-4-(2-(thiophen-2-yl)vinyl)aniline (*trans*-**3**).

**Figure S17.** Part of the ^1^H NMR spectrum (CDCl_3_) of (*E*)-*N*-(3-fluorobenzyl)-4-(2-(thiophen-2-yl)vinyl)aniline (*trans*-**3**).

**Figure S18.** ^13^C NMR spectrum (CDCl_3_) of (*E*)-*N*-(3-fluorobenzyl)-4-(2-(thiophen-2-yl)vinyl)aniline (*trans*-**3**).

**Figure S19.** ^1^H NMR spectrum (CDCl_3_) of (*Z*)-*N*-(4-chlorobenzyl)-4-(2-(thiophen-2-yl)vinyl)aniline (*cis*-**4**).

**Figure S20.** Part of the ^1^H NMR spectrum (CDCl_3_) of (*Z*)-*N*-(4-chlorobenzyl)-4-(2-(thiophen-2-yl)vinyl)aniline (*cis*-**4**).

**Figure S21.** ^13^C NMR spectrum (CDCl_3_) of (*Z*)-*N*-(4-chlorobenzyl)-4-(2-(thiophen-2-yl)vinyl)aniline (*cis*-**4**).

**Figure S22.** ^1^H NMR spectrum (CDCl_3_) of (*E*)-*N*-(4-chlorobenzyl)-4-(2-(thiophen-2-yl)vinyl)aniline (*trans*-**4**).

**Figure S23.** Part of the ^1^H NMR spectrum (CDCl_3_) of (*E*)-*N*-(4-chlorobenzyl)-4-(2-(thiophen-2-yl)vinyl)aniline (*trans*-**4**).

**Figure S24.** ^13^C NMR spectrum (CDCl_3_) of (*E*)-*N*-(4-chlorobenzyl)-4-(2-(thiophen-2-yl)vinyl)aniline (*trans*-**4**).

**Figure S25.** ^1^H NMR spectrum (CDCl_3_) of (*Z*)-*N*-(3-chlorobenzyl)-4-(2-(thiophen-2-yl)vinyl)aniline (*cis*-**5**).

**Figure S26.** Part of the ^1^H NMR spectrum (CDCl_3_) of (*Z*)-*N*-(3-chlorobenzyl)-4-(2-(thiophen-2-yl)vinyl)aniline (*cis*-**5**).

**Figure S27.** ^13^C NMR spectrum (CDCl_3_) of (*Z*)-*N*-(3-chlorobenzyl)-4-(2-(thiophen-2-yl)vinyl)aniline (*cis*-**5**).

 **Figure S28.** ^1^H NMR spectrum (CDCl_3_) of (*E*)-*N*-(3-chlorobenzyl)-4-(2-(thiophen-2-yl)vinyl)aniline (*trans*-**5**, with a minority of *cis*-**5**).

**Figure S29.** Part of the ^1^H NMR spectrum (CDCl_3_) of (*E*)-*N*-(3-chlorobenzyl)-4-(2-(thiophen-2-yl)vinyl)aniline (*trans*-**5**, with a minority of *cis*-**5**).

**Figure S30.** ^13^C NMR spectrum (CDCl_3_) of (*E*)-*N*-(3-chlorobenzyl)-4-(2-(thiophen-2-yl)vinyl)aniline (*trans*-**5**, with a minority of *cis*-**5**).

**Figure S31.** ^1^H NMR spectrum (CDCl_3_) of (*Z*)-*N*-(4-methoxybenzyl)-4-(2-(thiophen-2-yl)vinyl)aniline (*cis*-**6**).

**Figure S32.** Part of the ^1^H NMR spectrum (CDCl_3_) of (*Z*)-*N*-(4-methoxybenzyl)-4-(2-(thiophen-2-yl)vinyl)aniline (*cis*-**6**).

**Figure S33.** ^13^C NMR spectrum (CDCl_3_) of (*Z*)-*N*-(4-methoxybenzyl)-4-(2-(thiophen-2-yl)vinyl)aniline (*cis*-**6**).

**Figure S34.** ^1^H NMR spectrum (CDCl_3_) of (*E*)-*N*-(4-methoxybenzyl)-4-(2-(thiophen-2-yl)vinyl)aniline (*trans*-**6**).

**Figure S35.** Part of the ^1^H NMR spectrum (CDCl_3_) of (*E*)-*N*-(4-methoxybenzyl)-4-(2-(thiophen-2-yl)vinyl)aniline (*trans*-**6**).

**Figure S36.** ^13^C NMR spectrum (CDCl_3_) of (*E*)-*N*-(4-methoxybenzyl)-4-(2-(thiophen-2-yl)vinyl)aniline (*trans*-**6**).

**Figure S37.** ^1^H NMR spectrum (CDCl_3_) of mixture of *N*-(4-methoxybenzyl)-4-(2-(thiophen-2-yl)vinyl)aniline (isomers *cis*-**7** and *trans*-**7** and aldehyde).

**Figure S38.** Part of the ^1^H NMR spectrum (CDCl_3_) of mixture of *N*-(4-methoxybenzyl)-4-(2-(thiophen-2-yl)vinyl)aniline (isomers *cis*-**7** and *trans*-**7** and aldehyde).

**Figure S39.** ^1^H NMR spectrum (CDCl_3_) of (*Z*)-4-(2-(thiophen-2-yl)vinyl)-*N*-(thiophen-2-ylmethyl)aniline (*cis*-**8**).

**Figure S39.** Part of the ^1^H NMR spectrum (CDCl_3_) of (*Z*)-4-(2-(thiophen-2-yl)vinyl)-*N*-(thiophen-2-ylmethyl)aniline (*cis*-**8**).

**Figure S40.** ^13^C NMR spectrum (CDCl_3_) of (*Z*)-4-(2-(thiophen-2-yl)vinyl)-*N*-(thiophen-2-ylmethyl)aniline (*cis*-**8**).

**Figure S41.** ^1^H NMR spectrum (CDCl_3_) of (*E*)-4-(2-(thiophen-2-yl)vinyl)-*N*-(thiophen-2-ylmethyl)aniline (*trans*-**8**).

**Figure S42.** Part of the ^1^H NMR spectrum (CDCl_3_) of (*E*)-4-(2-(thiophen-2-yl)vinyl)-*N*-(thiophen-2-ylmethyl)aniline (*trans*-**8**).

**Figure S43.** ^13^C NMR spectrum (CDCl_3_) of (*E*)-4-(2-(thiophen-2-yl)vinyl)-*N*-(thiophen-2-ylmethyl)aniline (*trans*-**8**).

**Figure S44.** ^1^H NMR spectrum (CDCl_3_) of *N*-(4-fluorobenzyl)naphtho[2,1-*b*]thiophen-8-amine (**9**).

**Figure S45.** Part of the ^1^H NMR spectrum (CDCl_3_) of *N*-(4-fluorobenzyl)naphtho[2,1-*b*]thiophen-8-amine (**9**).

**Figure S46.** ^13^C NMR spectrum (CDCl_3_) of *N*-(4-fluorobenzyl)naphtho[2,1-*b*]thiophen-8-amine (**9**).

**Figure S47.** ^1^H NMR spectrum (CDCl_3_) of *N*-(3-fluorobenzyl)naphtho[2,1-*b*]thiophen-8-amine (**10**).

**Figure S48.** Part of the ^1^H NMR spectrum (CDCl_3_) of *N*-(3-fluorobenzyl)naphtho[2,1-*b*]thiophen-8-amine (**10**).

**Figure S49.** ^13^C NMR spectrum (CDCl_3_) of *N*-(3-fluorobenzyl)naphtho[2,1-*b*]thiophen-8-amine (**10**).

**Figure S50.** ^1^H NMR spectrum (CDCl_3_) of *N*-(4-chlorobenzyl)naphtho[2,1-*b*]thiophen-8-amine (**11**).

**Figure S51.** Part of the ^1^H NMR spectrum (CDCl_3_) of *N*-(4-chlorobenzyl)naphtho[2,1-*b*]thiophen-8-amine (**11**).

**Figure S52.** ^13^C NMR spectrum (CDCl_3_) of *N*-(4-chlorobenzyl)naphtho[2,1-*b*]thiophen-8-amine (**11**).

**Figure S53.** ^1^H NMR spectrum (CDCl_3_) of *N*-(3-chlorobenzyl)naphtho[2,1-*b*]thiophen-8-amine (**12**).

**Figure S54.** Part of the ^1^H NMR spectrum (CDCl_3_) of *N*-(3-chlorobenzyl)naphtho[2,1-*b*]thiophen-8-amine (**12**).

**Figure S55.** ^13^C NMR spectrum (CDCl_3_) of *N*-(3-chlorobenzyl)naphtho[2,1-*b*]thiophen-8-amine (**12**).

**Figure S56.** ^1^H NMR spectrum (CDCl_3_) of *N*-(4-methoxybenzyl)naphtho[2,1-*b*]thiophen-8-amine (**13**, with small amount of aldehyde).

**Figure S57.** Part of the ^1^H NMR spectrum (CDCl_3_) of *N*-(4-methoxybenzyl)naphtho[2,1-*b*]thiophen-8-amine (**13**, with small amount of aldehyde).

**Figure S58.** ^1^H NMR spectrum (CDCl_3_) of *N*-(3-methoxybenzyl)naphtho[2,1-*b*]thiophen-8-amine (**14**, with small amount of aldehyde).

**Figure S59.** Part of the ^1^H NMR spectrum (CDCl_3_) of *N*-(3-methoxybenzyl)naphtho[2,1-*b*]thiophen-8-amine (**14**, with small amount of aldehyde).

**Figure S60.** ^1^H NMR spectrum (CDCl_3_) of *N*-(thiophen-2-ylmethyl)naphtho[2,1-*b*]thiophen-8-amine (**15**).

**Figure S61.** Part of the ^1^H NMR spectrum (CDCl_3_) of *N*-(thiophen-2-ylmethyl)naphtho[2,1-*b*]thiophen-8-amine (**15**).

**Figure S62.** ^13^C NMR spectrum (CDCl_3_) of *N*-(thiophen-2-ylmethyl)naphtho[2,1-*b*]thiophen-8-amine (**15**).

**2. MS spectra and HRMS analyses of synthesized compounds 2-15**

**Figure S63.** Mass spectrum and result of the HRMS analysis for compound **2** as mixture of isomers.

**Figure S64.** Mass spectrum and result of the HRMS analysis for compound **3** as mixture of isomers.

**Figure S65.** Mass spectrum and result of the HRMS analysis for compound **4** and **5** as mixture of isomers.

**Figure S66.** Mass spectrum and result of the HRMS analysis for compound **6** as mixture of isomers.

**Figure S67.** Mass spectrum and result of the HRMS analysis for compound **7** as mixture of isomers.

**Figure S68.** Mass spectrum and result of the HRMS analysis for compound **8** as mixture of isomers.

**Figure S69.** Mass spectrum and result of the HRMS analysis for compound **9**.

**Figure S70.** Mass spectrum and result of the HRMS analysis for compound **10**.

**Figure S71.** Mass spectrum and result of the HRMS analysis for compound **11**.

**Figure S72.** Mass spectrum and result of the HRMS analysis for compound **12**.

**Figure S73.** Mass spectrum and result of the HRMS analysis for compound **15**.

**3. IR (ATR) spectra of the synthesized compounds 2-15**

**
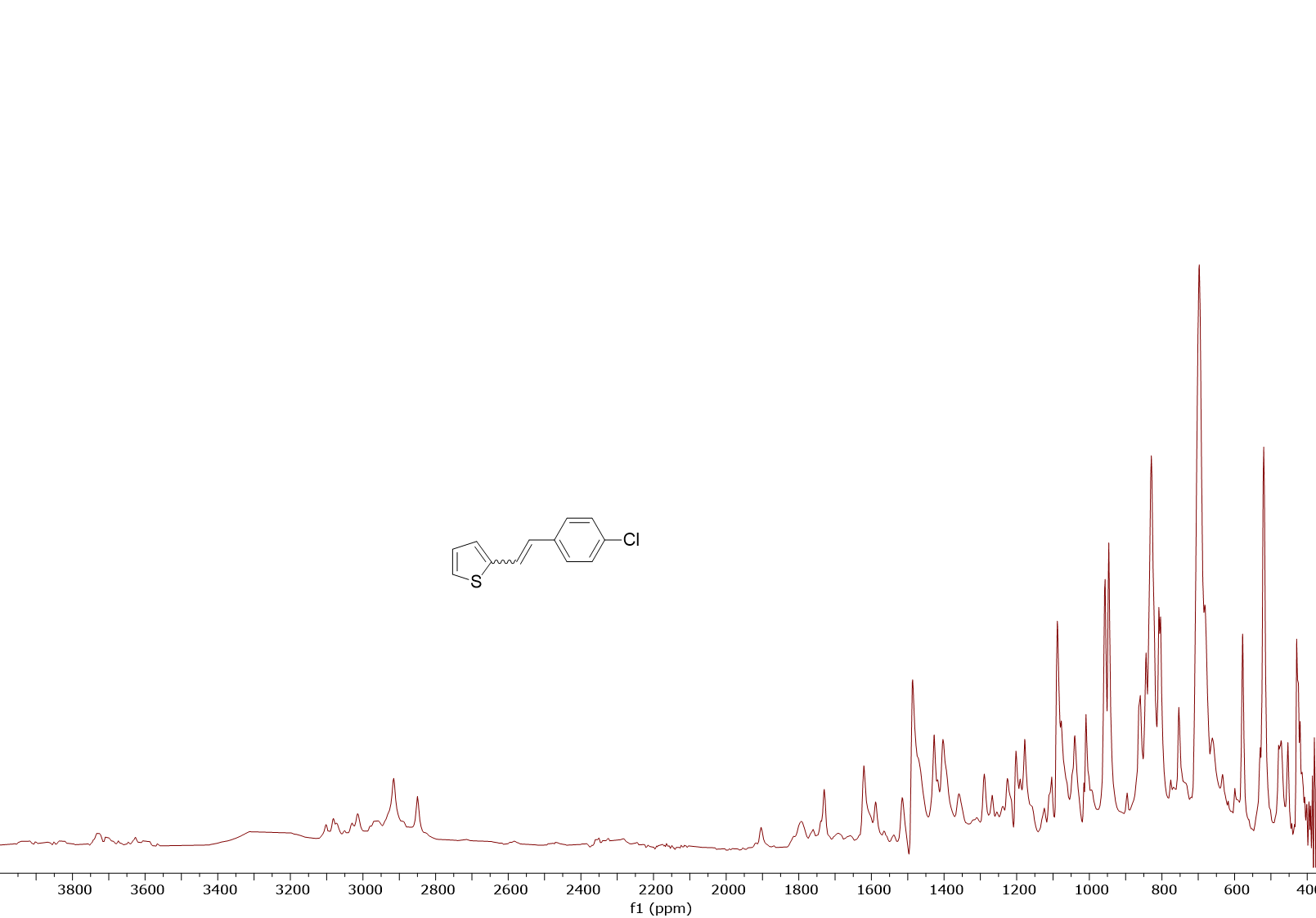
**
**Figure S74.** IR ART spectrum of 2-(4-chlorostyryl)thiophene (**1**).

 **Figure S75.** IR ART spectrum of *N*-(4-fluorobenzyl)-4-(2-(thiophen-2-yl)vinyl)aniline (**2**).

**Figure S76.** IR ART spectrum of *N*-(3-fluorobenzyl)-4-(2-(thiophen-2-yl)vinyl)aniline (**3**).

**Figure S77.** IR ART spectrum of (*Z*)-*N*-(4-methoxybenzyl)-4-(2-(thiophen-2-yl)vinyl)aniline (*cis*-**6**).

**Figure S78.** IR ART spectrum of *N*-(4-methoxybenzyl)-4-(2-(thiophen-2-yl)vinyl)aniline (isomers *cis*-**7** and *trans*-**7** and aldehyde).


**Figure S79.** IR ART spectrum of 4-(2-(thiophen-2-yl)vinyl)-*N*-(thiophen-2-ylmethyl)aniline (**8**).

**5. Cartesian coordinates of complexes of *cis*-8 and 15 with the active site of BChE**

cis-2

C 0.000000 0.000000 0.000000

C 0.000000 0.000000 1.371694

S 1.631636 0.000000 1.975352

C 2.289342 0.015158 0.385005

C 1.307995 0.013390 -0.562594

C -1.115505 -0.076020 2.316519

C -2.366623 0.390988 2.165404

C -2.930868 1.227810 1.092969

C -4.263844 1.057696 0.712692

C -4.854247 1.837398 -0.277067

C -4.120084 2.853132 -0.901587

C -2.784830 3.050737 -0.503431

C -2.207023 2.257451 0.468734

N -4.674062 3.694897 -1.859700

C -5.931913 3.354308 -2.498982

C -6.284694 4.383004 -3.546353

C -6.662362 3.998724 -4.832484

C -7.003655 4.947021 -5.793510

C -6.952939 6.287170 -5.446964

C -6.575623 6.706447 -4.178756

C -6.243507 5.745250 -3.231740

F -7.274363 7.209485 -6.365885

H -0.902079 -0.617977 3.235908

H -3.074744 0.121919 2.948225

H -4.855456 0.281803 1.192736

H -5.886042 1.652426 -0.554210

H -2.211469 3.853710 -0.960230

H -3.995526 4.091578 -2.499180

H -5.911130 2.349268 -2.951885

H -6.711539 3.336771 -1.726468

H -6.689821 2.943553 -5.090716

H -7.298334 4.665644 -6.798252

H -6.550335 7.766900 -3.953864

H -5.942824 6.047679 -2.232578

H -1.177823 2.438305 0.762785

H 1.507847 0.013324 -1.627550

H -0.914322 -0.019146 -0.581471

H 3.360319 0.025800 0.239383

cis-3

C 4.071672 1.092322 -0.767233

C 4.859335 0.107255 -0.228148

S 6.299828 0.796059 0.462285

C 5.820449 2.392381 0.033385

C 4.619149 2.397709 -0.613136

C 4.687991 -1.346611 -0.233270

C 3.541565 -2.047879 -0.223933

C 2.156475 -1.575410 -0.058956

C 1.118017 -2.235406 -0.719457

C -0.210627 -1.846089 -0.579878

C -0.545359 -0.784933 0.269887

C 0.491999 -0.133345 0.962287

C 1.808322 -0.519938 0.800465

N -1.860273 -0.386074 0.485942

C -2.913914 -0.821635 -0.410706

C -4.235888 -0.208309 -0.012713

C -5.077732 0.334802 -0.981316

C -6.296796 0.870187 -0.592304

C -6.708520 0.892888 0.730213

C -5.858639 0.352092 1.692153

C -4.632491 -0.195750 1.328042

H -7.668795 1.327126 0.984258

H 5.612292 -1.918007 -0.294291

H 3.639230 -3.125431 -0.350253

H 1.354631 -3.069330 -1.375954

H -0.979616 -2.375263 -1.131477

H 0.243786 0.674504 1.646337

H -1.952738 0.581994 0.770801

H -2.691480 -0.584474 -1.464018

H -2.990874 -1.914554 -0.341795

H -4.801483 0.352076 -2.030901

F -7.099086 1.391355 -1.532299

H -6.160078 0.357477 2.734805

H -3.971167 -0.617558 2.078628

H 2.587403 -0.004788 1.353752

H 4.141740 3.300060 -0.976296

H 3.133513 0.878646 -1.265923

H 6.450016 3.234910 0.282569

cis-4

C 0.000000 0.000000 0.000000

C 0.000000 0.000000 1.371631

S 1.631432 0.000000 1.975537

C 2.289211 0.015038 0.385250

C 1.308023 0.013276 -0.562525

C -1.116308 -0.075286 2.315658

C -2.364321 0.400228 2.166243

C -2.921966 1.247258 1.098223

C -4.256131 1.089478 0.717136

C -4.840725 1.880008 -0.267627

C -4.099112 2.894162 -0.885148

C -2.762372 3.078983 -0.486740

C -2.190220 2.275013 0.480084

N -4.646921 3.748022 -1.837769

C -5.905698 3.417806 -2.479432

C -6.262801 4.463133 -3.508698

C -6.678697 4.098403 -4.787885

C -7.027685 5.061881 -5.730626

C -6.949798 6.404396 -5.382502

C -6.531646 6.795429 -4.113879

C -6.191213 5.820669 -3.183767

Cl -7.380094 7.621760 -6.555322

H -0.906758 -0.623820 3.232028

H -3.075031 0.131389 2.946749

H -4.853629 0.315165 1.192349

H -5.873878 1.703956 -0.545473

H -2.183007 3.880452 -0.938580

H -3.963879 4.138101 -2.476770

H -5.888598 2.419720 -2.947517

H -6.684526 3.391150 -1.706380

H -6.730757 3.046841 -5.057058

H -7.350108 4.778000 -6.726316

H -6.478440 7.849372 -3.863672

H -5.861253 6.110590 -2.190194

H -1.159532 2.446030 0.774663

H 1.507947 0.012907 -1.627453

H -0.914294 -0.019269 -0.581522

H 3.360204 0.025254 0.239681

cis-5

C 0.000000 0.000000 0.000000

C 0.000000 0.000000 1.397538

C 1.213745 0.000000 2.082034

C 2.406440 -0.009541 1.366205

C 2.417966 -0.010133 -0.022869

C 1.201035 -0.003788 -0.700729

C -1.303969 -0.019269 2.160288

N -2.235536 0.940336 1.599403

C -3.490018 1.118372 2.175637

C -4.037111 0.206737 3.086224

C -5.318433 0.408399 3.590155

C -6.083587 1.524027 3.243657

C -5.529188 2.426715 2.320734

C -4.265242 2.230448 1.798884

C -7.444938 1.669053 3.786250

C -8.125496 2.783444 4.104124

C -7.639067 4.163855 4.134871

C -6.395380 4.656515 4.437631

C -6.335921 6.079214 4.426725

C -7.535374 6.654353 4.124038

S -8.752676 5.469226 3.848715

Cl 3.918539 -0.011734 2.236633

H 3.361327 -0.013653 -0.557481

H -9.158806 2.653454 4.420245

H -7.970130 0.727635 3.942783

H -5.728857 -0.318132 4.287338

H -3.473016 -0.663084 3.403705

H -3.863472 2.931665 1.071555

H -1.792860 1.805370 1.311541

H -1.115349 0.155572 3.232254

H -1.752188 -1.017763 2.076643

H 1.237281 0.009112 3.167462

H 1.195564 -0.004882 -1.786129

H -0.948713 0.004841 -0.528249

H -6.108839 3.288318 2.004759

H -5.439842 6.648370 4.643844

H -5.555498 4.012165 4.669916

H -7.772565 7.705999 4.045886

cis-6

C 0.000000 0.000000 0.000000

C 0.000000 0.000000 1.371678

S 1.631992 0.000000 1.974917

C 2.289533 0.014228 0.384494

C 1.307954 0.012960 -0.562822

C -1.114674 -0.077618 2.317277

C -2.367133 0.387209 2.168658

C -2.938252 1.224424 1.100908

C -4.277614 1.062050 0.739035

C -4.876901 1.842727 -0.244068

C -4.145526 2.853221 -0.881793

C -2.803163 3.042878 -0.502032

C -2.217023 2.248332 0.463957

N -4.708995 3.693091 -1.832081

C -5.978699 3.362121 -2.455046

C -6.326230 4.382966 -3.509540

C -6.640141 4.005055 -4.808832

C -6.973262 4.948057 -5.783635

C -6.984073 6.301158 -5.449569

C -6.662249 6.697344 -4.144718

C -6.339748 5.748441 -3.191554

O -7.288261 7.305474 -6.312137

C -7.621894 6.947524 -7.634472

H -0.899230 -0.620076 3.235871

H -3.072393 0.114907 2.953056

H -4.867570 0.291418 1.229641

H -5.913884 1.664917 -0.506012

H -2.231152 3.841152 -0.968631

H -4.041303 4.104216 -2.473280

H -5.971977 2.352125 -2.897338

H -6.749624 3.360376 -1.673360

H -6.627798 2.951739 -5.077691

H -7.212971 4.615693 -6.786640

H -6.677777 7.756623 -3.910796

H -6.088132 6.058319 -2.180703

H -1.182892 2.424213 0.743449

H 1.507543 0.012866 -1.627847

H -0.914457 -0.018376 -0.581300

H 3.360486 0.024479 0.238660

H -7.834165 7.880162 -8.156703

H -6.789688 6.433389 -8.130661

H -8.509949 6.304360 -7.661421

cis-7

C 0.000000 0.000000 0.000000

C 0.000000 0.000000 1.401497

C 1.206135 0.000000 2.086259

C 2.420932 -0.009276 1.390165

C 2.424640 -0.009515 -0.003858

C 1.205406 -0.003654 -0.685118

C -1.307062 -0.013741 2.158248

N -2.213834 0.984127 1.621600

C -3.454005 1.199541 2.207047

C -4.029730 0.290647 3.104144

C -5.298441 0.530222 3.621948

C -6.023907 1.680808 3.304168

C -5.442507 2.579981 2.394417

C -4.190684 2.347429 1.858808

C -7.374077 1.866087 3.861085

C -8.011441 2.998021 4.206340

C -7.476206 4.359416 4.261065

C -6.211371 4.802666 4.552715

C -6.104062 6.222402 4.576080

C -7.288373 6.845090 4.310166

S -8.549698 5.708494 4.028022

O 3.538151 -0.010229 2.163401

C 4.784645 -0.014013 1.504124

H 3.352730 -0.012271 -0.562849

H -9.046229 2.898912 4.528776

H -7.932726 0.941913 4.004621

H -5.731258 -0.194526 4.307506

H -3.496256 -0.606576 3.397405

H -3.767916 3.048096 1.142907

H -1.747074 1.829206 1.314744

H -1.120556 0.130329 3.234877

H -1.778411 -0.998996 2.046100

H 1.237941 0.009252 3.171806

H 1.209596 -0.004974 -1.770961

H -0.945126 0.003789 -0.534121

H -5.991566 3.468963 2.100128

H -5.185273 6.755384 4.790240

H -5.389629 4.124947 4.752489

H -7.490990 7.905737 4.262469

H 5.541906 -0.014598 2.287838

H 4.903758 -0.909031 0.881494

H 4.907921 0.878699 0.878910

cis-8

S 0.000000 0.000000 0.000000

C 0.000000 0.000000 1.734210

C 1.272458 0.000000 2.225517

C 2.267490 -0.008008 1.200385

C 1.724220 -0.007713 -0.048267

C -1.286871 -0.027465 2.499740

N -2.203836 0.975058 1.990986

C -3.441232 1.166270 2.597468

C -3.992212 0.241938 3.493006

C -5.256207 0.461507 4.032082

C -5.998238 1.606841 3.736265

C -5.441044 2.521715 2.827205

C -4.194930 2.308505 2.270441

C -7.341901 1.775240 4.315558

C -7.970217 2.899027 4.700724

C -7.427205 4.255849 4.784487

C -6.154293 4.683683 5.064025

C -6.038043 6.101444 5.125181

C -7.223649 6.737686 4.900064

S -8.497054 5.616603 4.610829

H -9.000704 2.795201 5.035150

H -7.900099 0.848176 4.440310

H -5.670339 -0.274515 4.717013

H -3.444673 -0.651627 3.771300

H -3.790992 3.019449 1.553980

H -1.743812 1.834725 1.714798

H -1.061073 0.105991 3.570896

H -1.762887 -1.010247 2.392725

H -6.004730 3.407291 2.550632

H -5.112152 6.623294 5.336025

H -5.333006 3.996218 5.229227

H -7.420858 7.800329 4.886069

H 2.237674 0.002000 -0.999580

H 3.334083 -0.004965 1.389808

H 1.491721 0.004886 3.287625

trans-2

C 0.000000 0.000000 0.000000

C 0.000000 0.000000 1.372175

S 1.631804 0.000000 1.977547

C 2.284958 0.001324 0.380311

C 1.306535 0.001017 -0.567749

C -1.166723 0.000467 2.236640

C -1.150601 0.011628 3.579704

C -2.308527 0.010982 4.473829

C -2.107725 0.075885 5.855789

C -3.164553 0.075997 6.759206

C -4.484803 0.020488 6.296304

C -4.698664 -0.049477 4.906705

C -3.638645 -0.056786 4.022690

N -5.580382 0.064601 7.150490

C -5.410560 -0.154851 8.575341

C -6.747526 -0.121393 9.276006

C -7.634773 0.934340 9.042823

C -8.864240 0.988508 9.688245

C -9.195123 -0.027664 10.574004

C -8.341618 -1.088711 10.827727

C -7.116147 -1.128231 10.167519

F -10.380080 0.018418 11.200106

H -2.113734 -0.006093 1.699937

H -0.184069 0.026653 4.085368

H 3.356356 0.002008 0.237013

H 1.506190 0.001522 -1.632540

H -1.090852 0.121255 6.238153

H -2.954178 0.115740 7.822066

H -5.718344 -0.092989 4.531899

H -3.849155 -0.118393 2.959159

H -6.412572 -0.375037 6.774953

H -4.896879 -1.105945 8.792223

H -4.771429 0.646589 8.967827

H -6.438307 -1.957718 10.350040

H -8.644156 -1.863534 11.523135

H -9.565114 1.798891 9.521438

H -7.353083 1.716543 8.343515

H -0.916256 -0.000097 -0.580017

trans-3

C 0.000000 0.000000 0.000000

C 0.000000 0.000000 1.394786

C 1.216090 0.000000 2.082088

C 2.395704 0.003976 1.356729

C 2.417085 0.009507 -0.030799

C 1.201443 0.008963 -0.706032

C -1.297782 0.019613 2.167353

N -1.250959 -0.921619 3.269714

C -2.358792 -1.097760 4.091177

C -3.432294 -0.199384 4.113667

C -4.491843 -0.400096 4.991295

C -4.539065 -1.489912 5.865510

C -3.461453 -2.392299 5.822661

C -2.398555 -2.202150 4.962850

C -5.684798 -1.641276 6.762633

C -5.856271 -2.587023 7.700820

C -7.005594 -2.715429 8.578944

C -7.187804 -3.665149 9.552456

C -8.420397 -3.530102 10.253653

C -9.161306 -2.478815 9.804208

S -8.371237 -1.637943 8.521128

F 3.561238 0.003184 2.020080

H -5.092191 -3.345877 7.859751

H -6.464784 -0.888905 6.637449

H -10.129469 -2.145263 10.150381

H -8.738338 -4.186091 11.055050

H -5.313598 0.311942 4.992992

H -3.449798 0.656015 3.447692

H -1.572648 -2.909217 4.956061

H -3.455114 -3.262144 6.472680

H -0.768611 -1.783031 3.040837

H -2.141381 -0.175609 1.485263

H -1.452495 1.023583 2.583534

H -0.944619 -0.009483 -0.535970

H 1.191142 0.007864 -1.791244

H 3.368332 0.011221 -0.550796

H 1.247112 -0.003146 3.166827

H -6.452778 -4.436188 9.755326

trans-4

C 0.000000 0.000000 0.000000

C 0.000000 0.000000 1.372176

S 1.631706 0.000000 1.977673

C 2.284862 0.001264 0.380512

C 1.306493 0.001048 -0.567661

C -1.166837 0.000149 2.236473

C -1.151000 0.011322 3.579487

C -2.309573 0.009557 4.472994

C -2.109712 0.079550 5.854749

C -3.167188 0.078404 6.757585

C -4.486579 0.016343 6.293959

C -4.699667 -0.058735 4.904794

C -3.639136 -0.064888 4.021276

N -5.584354 0.058479 7.147362

C -5.412051 -0.159324 8.571612

C -6.746568 -0.121049 9.276718

C -7.649348 0.913793 9.016714

C -8.874965 0.971566 9.669307

C -9.197305 -0.017621 10.593954

C -8.317744 -1.057051 10.869070

C -7.096152 -1.102404 10.202638

Cl -10.733249 0.047137 11.417624

H -2.113776 -0.006629 1.699635

H -0.184751 0.027274 4.085605

H 3.356268 0.001865 0.237251

H 1.506257 0.001519 -1.632418

H -1.093296 0.129971 6.237604

H -2.957087 0.122000 7.820351

H -5.718955 -0.107491 4.529577

H -3.848854 -0.131274 2.957907

H -6.410788 -0.392613 6.772240

H -4.898897 -1.110192 8.790609

H -4.772742 0.642660 8.962763

H -6.407567 -1.917594 10.408142

H -8.590040 -1.821206 11.588730

H -9.576811 1.774024 9.469998

H -7.384311 1.678050 8.291567

H -0.916204 -0.000107 -0.580096

trans-5

C 0.000000 0.000000 0.000000

C 0.000000 0.000000 1.372147

S 1.631809 0.000000 1.977650

C 2.284816 0.001174 0.380533

C 1.306484 0.000863 -0.567637

C -1.166626 0.000679 2.236729

C -1.150145 0.010672 3.579754

C -2.308128 0.011613 4.473969

C -2.106850 0.068395 5.856041

C -3.163854 0.070242 6.759486

C -4.483923 0.025901 6.296007

C -4.698609 -0.036247 4.906503

C -3.638640 -0.045843 4.022429

N -5.581222 0.074407 7.149635

C -5.413418 -0.151233 8.572274

C -6.746121 -0.086249 9.280854

C -7.636520 0.948807 8.984594

C -8.852313 1.015533 9.651892

C -9.207487 0.075101 10.614301

C -8.316027 -0.952163 10.903693

C -7.094745 -1.036186 10.239537

Cl -9.959535 2.310749 9.275497

H -2.113732 -0.004660 1.700188

H -0.183599 0.023677 4.085367

H 3.356213 0.001830 0.237217

H 1.506230 0.001346 -1.632401

H -1.089775 0.105868 6.238638

H -2.952597 0.103333 7.822407

H -5.718587 -0.070366 4.531594

H -3.849662 -0.100063 2.958633

H -6.414048 -0.361430 6.770608

H -4.921048 -1.113202 8.790107

H -4.758583 0.636462 8.966678

H -6.408072 -1.847136 10.465395

H -8.580888 -1.696205 11.648109

H -10.164075 0.150853 11.119161

H -7.378338 1.692071 8.236820

H -0.916257 0.000150 -0.580017

trans-6

C 0.000000 0.000000 0.000000

C 0.000000 0.000000 1.372184

S 1.632048 0.000000 1.977192

C 2.285178 0.002408 0.379774

C 1.306514 0.001740 -0.567957

C -1.166163 0.000531 2.237495

C -1.148580 0.017540 3.580610

C -2.304418 0.017654 4.476892

C -2.101202 0.092989 5.858113

C -3.155752 0.095834 6.763727

C -4.477634 0.032131 6.304346

C -4.693509 -0.049155 4.915094

C -3.635336 -0.058587 4.029114

N -5.568269 0.078461 7.160553

C -5.399962 -0.131562 8.588023

C -6.739431 -0.116601 9.282943

C -7.632967 0.931378 9.070506

C -8.871554 0.970730 9.707565

C -9.227710 -0.059372 10.580917

C -8.341104 -1.117587 10.801764

C -7.115355 -1.141938 10.155075

O -10.405673 -0.123482 11.254633

C -11.326395 0.926184 11.057415

H -2.113744 -0.010724 1.701886

H -0.181243 0.038205 4.084685

H 3.356560 0.003361 0.236384

H 1.505841 0.002446 -1.632841

H -1.083575 0.145079 6.237846

H -2.944267 0.144912 7.825954

H -5.713766 -0.100441 4.542781

H -3.847577 -0.129429 2.966431

H -6.408504 -0.348371 6.788976

H -4.874193 -1.075492 8.807450

H -4.770291 0.678972 8.977613

H -6.436639 -1.973058 10.329169

H -8.642035 -1.909248 11.479599

H -9.541399 1.801492 9.519401

H -7.355685 1.732089 8.389823

H -0.916365 -0.000335 -0.579839

H -12.191452 0.690701 11.676981

H -10.906062 1.889789 11.369931

H -11.637455 0.992280 10.007708

trans-7

C 0.000000 0.000000 0.000000

C 0.000000 0.000000 1.372180

S 1.632027 0.000000 1.977375

C 2.285046 0.000301 0.379990

C 1.306460 0.000198 -0.567861

C -1.166473 -0.000255 2.236996

C -1.149687 -0.000517 3.580139

C -2.306515 -0.001205 4.475431

C -2.103184 0.015744 5.858523

C -3.158682 0.010739 6.763228

C -4.480697 -0.000106 6.301235

C -4.697027 -0.019983 4.910146

C -3.638310 -0.022713 4.024609

N -5.572678 0.042582 7.158316

C -5.409341 -0.235290 8.574306

C -6.745546 -0.200199 9.277820

C -7.602694 0.886223 9.059470

C -8.831300 0.941485 9.713722

C -9.207599 -0.087045 10.587544

C -8.354841 -1.156823 10.795847

C -7.122347 -1.221768 10.140632

O -9.730459 1.949281 9.570365

C -9.386653 3.010516 8.707044

H -2.113741 0.000520 1.700730

H -0.182661 0.001207 4.085129

H 3.356435 0.000684 0.236599

H 1.505894 0.000366 -1.632703

H -1.085138 0.025684 6.240422

H -2.947033 0.011712 7.826575

H -5.717780 -0.027954 4.535737

H -3.851245 -0.042136 2.959916

H -6.419002 -0.356147 6.768895

H -4.917242 -1.204975 8.752986

H -4.754688 0.537082 8.998774

H -6.459554 -2.066877 10.301535

H -8.651312 -1.954287 11.470180

H -10.170565 -0.016876 11.081890

H -7.287097 1.667236 8.376274

H -0.916332 -0.000280 -0.579886

H -10.225095 3.706154 8.733816

H -8.477255 3.521271 9.045640

H -9.238503 2.656978 7.679539

trans-8

S 0.000000 0.000000 0.000000

C 0.000000 0.000000 1.734291

C 1.272374 0.000000 2.225693

C 2.267445 -0.008330 1.200594

C 1.724246 -0.008051 -0.048076

C -1.286945 -0.027896 2.499645

N -2.204189 0.974275 1.990671

C -3.445034 1.159205 2.590195

C -3.995718 0.235699 3.486616

C -5.261463 0.451045 4.020285

C -6.020951 1.581156 3.703945

C -5.452772 2.504534 2.808358

C -4.201030 2.300663 2.263767

C -7.342680 1.749168 4.308203

C -8.187124 2.778705 4.131705

C -9.496636 2.918717 4.743363

C -10.368172 3.965477 4.577391

C -11.580578 3.816498 5.310334

C -11.616336 2.657275 6.025215

S -10.176201 1.729619 5.817472

H -7.912368 3.607992 3.482313

H -7.648591 0.936512 4.968581

H -12.405969 2.289093 6.665031

H -12.387069 4.539808 5.303918

H -5.669750 -0.280360 4.713543

H -3.442633 -0.651050 3.775326

H -3.788794 3.023073 1.563713

H -6.002061 3.398440 2.528397

H -1.745424 1.835324 1.716648

H -1.061568 0.105854 3.570773

H -1.763009 -1.010603 2.392385

H -10.139289 4.816008 3.944809

H 2.237743 0.001555 -0.999387

H 3.333999 -0.005619 1.390185

H 1.491748 0.005092 3.287773

Molecule 9

C 0.348684 0.716457 0.058966

C 0.158028 0.103306 1.296818

C 1.274109 -0.302647 2.031118

C 2.562897 -0.090704 1.553110

C 2.717159 0.527001 0.321025

C 1.630763 0.932115 -0.438158

C -1.233453 -0.107349 1.869000

H -1.247302 -1.046844 2.444052

N -2.284624 -0.081670 0.871796

C -2.579319 -1.209102 0.108644

C -3.802412 -1.310093 -0.536455

C -4.120066 -2.422053 -1.338098

C -3.168719 -3.468037 -1.490656

C -1.928456 -3.343248 -0.825098

C -1.629461 -2.252772 -0.046713

C -3.471292 -4.608009 -2.296972

C -4.668757 -4.732289 -2.939758

C -5.620966 -3.691556 -2.789574

C -5.379188 -2.556962 -2.016368

C -6.498137 -1.654903 -2.027271

C -7.529863 -2.108044 -2.781590

S -7.199902 -3.644586 -3.514753

H -1.447228 0.697989 2.579957

H -2.718358 -5.386111 -2.389894

H -4.893671 -5.600196 -3.550767

H -1.194256 -4.136816 -0.938861

H -0.661544 -2.182068 0.437185

H -4.529017 -0.509925 -0.418349

H -8.481950 -1.626646 -2.957133

H -3.101963 0.448139 1.139954

H -6.527897 -0.711094 -1.496082

H 1.136023 -0.794328 2.991480

H 3.440661 -0.400089 2.109446

F 3.956291 0.729867 -0.152823

H 1.799547 1.401758 -1.400897

H -0.520713 1.012850 -0.519304

Molecule 10

C -0.005063 0.009198 -0.001459

C -0.005406 0.001772 1.395254

C 1.205122 -0.008113 2.083692

C 2.386214 -0.019832 1.357801

C 2.411128 -0.021771 -0.028338

C 1.195162 -0.006780 -0.706100

C -1.325924 -0.022901 2.146179

N -1.227223 0.430314 3.517930

C -1.237969 1.788958 3.826943

C -0.915363 2.761543 2.844494

C -0.905061 4.093928 3.174729

C -1.207426 4.541729 4.480414

C -1.527297 3.568085 5.466277

C -1.536973 2.205514 5.114894

C -1.195091 5.930797 4.814217

C -1.486813 6.366486 6.074220

C -1.806534 5.398546 7.060721

C -1.833378 4.030924 6.791230

C -2.182524 3.263893 7.955464

C -2.406050 4.038170 9.046015

S -2.204996 5.729455 8.719936

H 3.363123 -0.027549 -0.546909

H -2.069981 0.559007 1.579914

H -1.689005 -1.055890 2.172538

H -0.944903 6.644632 4.033962

H -1.475772 7.422622 6.322573

H -0.655630 4.831279 2.415944

H -0.662295 2.452316 1.836224

H -1.781805 1.460018 5.867467

H -2.680707 3.715473 10.040733

H -1.728154 -0.139656 4.184846

H -2.261065 2.183480 7.974986

H -0.949882 0.029747 -0.538772

H 1.186657 -0.000654 -1.791467

F 3.550786 -0.027807 2.023752

H 1.228852 0.006730 3.167807

Molecule 11

C -0.017710 -0.049358 0.001003

C -0.015380 0.001532 1.395934

C 1.204709 0.039204 2.067997

C 2.405434 0.016795 1.363914

C 2.377828 -0.045824 -0.024046

C 1.172360 -0.078232 -0.717229

C -1.335653 -0.008980 2.147118

N -1.231400 0.440774 3.519966

C -1.239209 1.798790 3.833213

C -0.907891 2.773684 2.856067

C -0.893108 4.104843 3.191502

C -1.199960 4.548873 4.497410

C -1.529343 3.572880 5.477836

C -1.542626 2.211689 5.121258

C -1.183604 5.936682 4.836647

C -1.481174 6.368673 6.096530

C -1.811427 5.398398 7.077303

C -1.842089 4.031929 6.802520

C -2.203142 3.262020 7.961191

C -2.431711 4.033262 9.052841

S -2.221033 5.724883 8.734678

Cl 3.878684 -0.075504 -0.914393

H -2.073728 0.583697 1.583773

H -1.713180 -1.036881 2.172057

H -0.925955 6.652492 4.060660

H -1.467317 7.423825 6.348929

H -0.637092 4.843926 2.436615

H -0.651881 2.467298 1.847700

H -1.794971 1.464489 5.869677

H -2.715357 3.708057 10.044217

H -1.735242 -0.128733 4.185167

H -2.286685 2.181910 7.976235

H -0.963458 -0.065960 -0.536098

H 1.171438 -0.118847 -1.800952

H 3.356555 0.050944 1.884132

H 1.207138 0.097407 3.151847

Molecule 12

C -0.008538 -0.047442 0.007291

C -0.001713 0.003365 1.401913

C 1.214038 0.041031 2.081558

C 2.412265 0.018946 1.372309

C 2.415776 -0.042188 -0.017587

C 1.195552 -0.074676 -0.684147

C -1.328276 -0.007667 2.143839

N -1.233155 0.441610 3.516668

C -1.239289 1.799186 3.830894

C -0.907258 2.774518 2.854436

C -0.892810 4.105509 3.190649

C -1.199858 4.548752 4.496769

C -1.528601 3.572171 5.476817

C -1.542177 2.211224 5.119353

C -1.184069 5.936420 4.836547

C -1.480995 6.367749 6.096801

C -1.809695 5.396827 7.077479

C -1.840048 4.030474 6.802132

C -2.199076 3.259930 7.961066

C -2.426337 4.030670 9.053394

S -2.217633 5.722535 8.735351

H 3.342186 -0.058285 -0.580707

H -2.062287 0.584300 1.574772

H -1.703729 -1.036281 2.164714

H -0.927555 6.652622 4.060543

H -1.467861 7.422799 6.349617

H -0.637160 4.845059 2.436115

H -0.651356 2.469172 1.845694

H -1.794477 1.463460 5.867211

H -2.708448 3.705049 10.045056

H -1.738331 -0.128966 4.179803

H -2.282104 2.179775 7.975730

H -0.945673 -0.064987 -0.542546

Cl 1.173569 -0.141909 -2.428759

H 3.356813 0.052875 1.906174

H 1.208316 0.098633 3.164992

Molecule 13

C 0.017552 -0.004973 0.038492

C -0.017609 -0.003311 1.437519

C 1.187061 -0.009367 2.129448

C 2.409389 -0.025628 1.455912

C 2.427733 -0.037795 0.061669

C 1.220582 -0.026637 -0.646476

C -1.353378 -0.017612 2.158803

N -1.279686 0.438324 3.533432

C -1.271734 1.794606 3.843996

C -0.920126 2.764340 2.867740

C -0.893205 4.095650 3.200547

C -1.206609 4.546845 4.502664

C -1.555727 3.576859 5.482247

C -1.581764 2.215220 5.128770

C -1.177491 5.934956 4.838779

C -1.480273 6.374156 6.095136

C -1.828985 5.409980 7.075447

C -1.873236 4.043350 6.803436

C -2.251173 3.280352 7.961506

C -2.478369 4.056345 9.050044

S -2.246626 5.744947 8.729340

O 3.557068 -0.051973 -0.695942

H -2.081989 0.572510 1.580023

H -1.734163 -1.044534 2.184428

H -0.905605 6.645697 4.062874

H -1.456921 7.429766 6.344849

H -0.622104 4.830077 2.446261

H -0.657342 2.451149 1.863422

H -1.849307 1.472745 5.876658

H -2.772559 3.736640 10.040119

H -1.801637 -0.123824 4.190696

H -2.346996 2.201271 7.977863

H -0.914624 0.013503 -0.522497

H 1.258662 -0.026606 -1.730700

H 3.330175 -0.022882 2.027348

H 1.169021 0.011852 3.214886

C 4.792303 -0.050667 -0.016868

H 5.562578 -0.061614 -0.787874

H 4.907529 0.848546 0.600587

H 4.899975 -0.938630 0.618075

Molecule 14

C -0.043743 -0.004901 -0.002604

C -0.056080 -0.003039 1.389039

C 1.144545 -0.002676 2.101177

C 2.357147 -0.009548 1.411746

C 2.371488 -0.024846 0.012675

C 1.173651 -0.022573 -0.684096

C -1.385948 -0.024661 2.124820

N -1.305203 0.425311 3.500486

C -1.284629 1.781556 3.814904

C -0.927817 2.750836 2.840316

C -0.886170 4.080642 3.178165

C -1.188613 4.529904 4.483553

C -1.542775 3.559949 5.461345

C -1.584065 2.199918 5.102695

C -1.144007 5.916486 4.824549

C -1.436219 6.353739 6.084033

C -1.789354 5.389459 7.062636

C -1.848609 4.024274 6.785983

C -2.227578 3.260950 7.943452

C -2.440910 4.035368 9.035961

S -2.194594 5.722668 8.719814

H 3.330064 -0.028065 -0.494951

H -2.121040 0.561543 1.551210

H -1.755079 -1.055612 2.147440

H -0.869180 6.627485 4.049975

H -1.401282 7.408123 6.337533

H -0.611946 4.815264 2.425243

H -0.675132 2.439683 1.832632

H -1.855405 1.457188 5.849008

H -2.733025 3.715167 10.026493

H -1.835071 -0.132691 4.155126

H -2.334407 2.182860 7.956653

H -0.980457 0.008322 -0.554029

H 1.186228 -0.025654 -1.769848

O 3.575015 0.001180 2.013844

H 1.101146 0.018322 3.183798

C 3.602418 0.058517 3.423234

H 4.655209 0.081977 3.704218

H 3.103376 0.961262 3.795014

H 3.126276 -0.823881 3.867648

Molecule 15

S -0.078224 -0.014454 0.025308

C -0.037243 0.024203 1.762655

C 1.271023 0.002992 2.244105

C 2.232243 -0.045890 1.176546

C 1.653853 -0.059410 -0.049796

C 1.500292 0.029839 3.661856

C 0.365325 0.077332 4.517130

C -0.953123 0.096083 3.967184

C -1.166804 0.070875 2.619443

C 2.790024 0.016842 4.224943

C 2.979175 0.045863 5.597379

C 1.845876 0.089467 6.450829

C 0.582981 0.104687 5.913173

N 4.270739 0.003789 6.122808

C 4.543909 0.455648 7.474450

C 4.231320 -0.590297 8.511178

H 3.989743 1.373857 7.724448

H 5.610136 0.701330 7.518949

H -1.795424 0.131372 4.652957

H -2.170286 0.085426 2.207052

H -0.279483 0.136153 6.574138

H 1.977520 0.092401 7.527431

H 3.658966 -0.019505 3.572520

H 2.145507 -0.094910 -1.012102

H 4.984183 0.254501 5.452467

H 3.305702 -0.071596 1.320955

C 3.859578 -0.408048 9.811625

C 3.696836 -1.638266 10.519941

C 3.943531 -2.722933 9.733660

S 4.372061 -2.270325 8.125436

H 3.878169 -3.768365 10.001182

H 3.399579 -1.705465 11.559673

H 3.706106 0.572559 10.249872

Structure of the complex between compound *cis*-**8** and the active site of AChE

C 0.889961 38.226952 30.295956

C -3.332988 37.031044 35.064047

C 2.534988 36.280991 40.809956

C 6.338011 31.711026 34.739033

C 6.231986 28.995004 32.101893

C 5.773016 33.834019 29.148060

C 11.481997 27.166997 40.006992

C 9.329009 25.833998 37.179019

C 10.900991 27.559997 34.151027

C -1.752009 32.065003 22.959025

C 0.734010 23.002996 30.565981

C 3.590064 27.408976 26.558933

C 1.828003 19.981009 40.815001

C -2.983051 25.207947 38.936079

C -7.343984 27.354048 34.761006

C 2.985996 24.173990 42.399991

H 1.902271 38.601136 30.108236

H 0.730932 38.234697 31.380921

H 0.183771 38.943740 29.861011

C 0.698049 36.828651 29.708644

H 0.927951 36.846563 28.623606

H 1.446364 36.124172 30.150354

C -0.690843 36.301754 29.948193

C -1.603189 36.189637 28.889305

H -1.306537 36.487716 27.882990

C -1.088783 35.921816 31.243584

H -0.382136 36.006269 32.070292

C -2.894780 35.705365 29.101920

H -3.611809 35.630672 28.289997

C -2.372967 35.445890 31.483662

H -2.697973 35.165254 32.485247

C -3.275721 35.340839 30.405973

O -4.536125 34.869520 30.565084

H -4.830042 34.851602 31.603743

H -3.046579 37.272223 36.094806

H -3.232699 37.950112 34.472824

H -2.600965 36.309570 34.678158

C -4.751602 36.476492 35.006443

H -5.468995 37.192293 35.452141

H -4.830991 35.547675 35.609949

C -5.136558 36.202644 33.562080

O -5.547191 37.099504 32.816508

O -5.008791 34.980521 33.147136

H 3.210088 37.128069 40.970827

H 1.552545 36.554539 41.212185

H 2.417739 36.151723 39.727549

C 3.073743 35.009831 41.468902

H 4.080675 34.775887 41.062475

H 3.226776 35.186452 42.555163

C 2.158262 33.854284 41.275369

C 2.325863 32.802654 40.383430

H 3.125852 32.623464 39.683871

C 0.924007 33.622545 41.986599

N 1.243032 31.907164 40.500090

H 1.128949 31.056633 39.975494

C 0.372059 32.393633 41.482081

C 0.229695 34.320772 42.993432

H 0.637997 35.251671 43.381982

C -0.845520 31.869147 41.968792

H -1.244774 30.938818 41.575054

C -0.962293 33.801852 43.465758

H -1.516067 34.323568 44.243425

C -1.494130 32.585661 42.955973

H -2.437045 32.220452 43.362484

H 6.774538 32.721973 34.765944

H 6.770112 31.163705 35.592663

H 5.262918 31.827502 34.949314

C 6.596496 31.044672 33.433674

O 7.193354 31.572714 32.492674

N 6.103201 29.757798 33.346953

H 5.599281 29.311350 34.110243

H 6.649722 27.991139 32.297697

H 6.902559 29.504163 31.384025

H 5.249651 28.862754 31.608139

H 6.465504 34.578385 28.739556

H 5.158582 33.462181 28.320078

H 6.375460 32.991443 29.508683

C 4.911349 34.433402 30.259531

H 4.427460 35.361506 29.892322

H 5.559408 34.756785 31.102700

C 3.881088 33.465825 30.776634

C 2.530978 33.830276 30.857454

H 2.207895 34.829416 30.533613

C 4.276563 32.185138 31.207830

H 5.334204 31.902883 31.167257

C 1.572840 32.939938 31.351792

H 0.528695 33.233005 31.424374

C 3.344497 31.277206 31.694792

H 3.636583 30.283224 32.028113

C 1.993991 31.667342 31.758929

O 1.135485 30.710889 32.232318

H 0.192120 31.060319 32.360500

H 12.568745 27.239132 39.773418

H 11.368216 26.195657 40.533829

C 10.718219 27.226431 38.720738

O 10.538424 28.252550 38.066809

C 11.086846 28.309687 40.958145

H 10.939805 29.244819 40.377389

H 11.935129 28.509230 41.643228

C 9.836770 28.007009 41.783508

H 9.893240 27.001073 42.234714

H 9.763230 28.718411 42.626245

C 8.557705 28.114211 40.937376

O 8.037612 29.244900 40.796251

O 8.092789 27.056718 40.446316

N 10.317411 25.972262 38.254300

H 10.368888 25.165085 38.883200

H 8.927473 24.773138 37.214570

C 9.963695 25.990833 35.798474

O 10.017746 25.051574 35.003685

C 8.106682 26.773347 37.373033

H 7.873797 26.890037 38.460154

H 8.255781 27.771152 36.926082

O 6.989470 26.227398 36.677059

H 6.544811 25.522152 37.242560

N 10.466316 27.234914 35.513344

H 10.402847 28.012295 36.194397

H 11.929296 27.990826 34.194872

H 10.975169 26.627771 33.539769

C 9.946030 28.550874 33.485136

H 10.280285 28.796635 32.470327

H 9.875076 29.493156 34.042388

H 8.928768 28.147048 33.405522

H -2.142832 31.764816 21.981229

H -0.730335 31.675465 23.043401

H -1.683591 33.158717 22.968910

C -2.639093 31.549580 24.094265

H -2.725450 30.443575 24.028980

H -3.672083 31.935935 23.968514

C -2.102201 31.930130 25.427556

C -2.578617 32.934485 26.260883

H -3.406878 33.606290 26.112230

C -0.975682 31.319125 26.090579

N -1.796007 32.987095 27.432039

H -1.935070 33.625496 28.201473

C -0.805441 32.001891 27.344591

C -0.097815 30.267679 25.759561

H -0.219440 29.741265 24.815269

C 0.220327 31.652701 28.250600

H 0.335270 32.183039 29.193073

C 0.900947 29.929726 26.652715

H 1.593857 29.111268 26.424986

C 1.058635 30.618631 27.886058

H 1.868192 30.305571 28.557472

H 0.483456 22.250090 29.810943

H 1.809782 23.204614 30.489981

H 0.213018 23.930605 30.299197

C 0.350045 22.531319 31.967971

H 0.872497 21.579950 32.200966

H -0.730850 22.282867 31.997516

C 0.681808 23.571665 33.004924

C 1.983137 23.659249 33.524807

H 2.755179 22.974825 33.176988

C -0.303826 24.461552 33.459230

H -1.314534 24.403275 33.060426

C 2.291346 24.623344 34.487738

H 3.303363 24.685888 34.889614

C 0.008748 25.422874 34.424558

H -0.761086 26.106815 34.781943

C 1.305844 25.507628 34.939270

H 1.549396 26.259442 35.688531

H 4.110846 27.585045 25.611154

H 2.663962 28.009927 26.541752

H 3.294912 26.353525 26.584480

C 4.472242 27.776658 27.751648

H 5.458252 27.275214 27.658406

H 4.698841 28.863071 27.730399

C 3.820652 27.400259 29.056165

C 3.027740 28.327059 29.748309

H 2.876297 29.332793 29.341653

C 3.999610 26.113670 29.590069

H 4.613342 25.386647 29.062158

C 2.416226 27.972651 30.954190

H 1.794484 28.702058 31.483144

C 3.391230 25.764843 30.798292

H 3.534817 24.767001 31.213869

C 2.596900 26.691612 31.482028

H 2.121913 26.410837 32.422949

H 1.127485 20.159707 39.993460

H 2.498560 20.859419 40.878715

H 1.253247 19.957943 41.746759

C 2.618040 18.691422 40.607700

H 3.007584 18.652360 39.570213

H 1.944133 17.818546 40.708636

C 3.781561 18.550127 41.595822

H 3.438550 18.728701 42.629851

H 4.181715 17.521274 41.572462

C 4.895350 19.536566 41.241939

O 4.928353 20.633078 41.873877

O 5.724413 19.228637 40.358822

H -3.375695 24.184976 38.951705

H -3.778379 25.874500 39.289746

H -2.166250 25.258125 39.666614

C -2.505057 25.595289 37.537067

H -1.734629 24.875321 37.190171

H -3.339394 25.497620 36.811785

C -1.962569 26.999250 37.519336

C -2.837437 28.091927 37.414340

H -3.910284 27.923332 37.339361

C -0.581728 27.228771 37.616669

H 0.105704 26.388046 37.704031

C -2.336300 29.395714 37.408014

H -3.023164 30.239445 37.329063

C -0.084332 28.534573 37.606703

H 0.989972 28.705394 37.679903

C -0.959033 29.620664 37.503662

H -0.569555 30.637197 37.499908

H -7.475608 26.274269 34.632271

H -8.056076 27.856815 34.096249

H -7.629462 27.602921 35.789715

C -5.905844 27.776684 34.459060

C -5.701223 29.259118 34.622336

C -5.634739 29.825019 35.907259

H -5.756833 29.187835 36.783765

C -5.559446 30.088097 33.498554

H -5.606748 29.658904 32.495952

C -5.413225 31.188814 36.078919

H -5.354426 31.634776 37.067421

C -5.343075 31.458511 33.641991

H -5.232829 32.100722 32.771473

C -5.265977 31.999619 34.936630

O -5.031931 33.317226 35.161028

H -4.965612 33.890694 34.253843

H 2.072431 24.024764 42.985414

H 3.141111 25.254492 42.292855

H 3.825669 23.784532 42.988835

C 2.889287 23.484147 41.040073

H 2.707065 22.385806 41.184618

H 2.003615 23.862253 40.486337

C 4.109817 23.682392 40.216878

N 5.219761 22.829650 40.289706

H 5.299731 21.981911 40.913867

C 4.415193 24.644627 39.243736

H 3.817074 25.471290 38.911033

C 6.154247 23.294334 39.359636

H 7.113860 22.828758 39.192045

N 5.681975 24.394788 38.713236

H -5.632901 27.459299 33.430907

H -5.206343 27.228206 35.124803

C -1.072141 27.032904 28.962046

C -2.132626 26.266803 28.581089

S -1.650784 25.237798 27.227464

C -0.024616 25.877714 27.200531

C 0.124092 26.807275 28.177097

C -3.489736 26.157155 29.060891

C -4.069600 26.922794 29.998271

C -3.448940 28.033069 30.740650

C -2.761952 27.782343 31.938801

C -2.228055 28.834190 32.680940

C -2.380249 30.160196 32.229686

C -3.055928 30.412933 31.017113

C -3.581946 29.353187 30.281561

N -1.764069 31.255735 32.925392

C -1.753647 31.139216 34.422405

C -1.244246 32.392462 35.026389

S 0.472360 32.755171 34.995137

C 0.262238 34.238925 35.892389

C -1.051690 34.430592 36.176317

C -1.914901 33.380014 35.678060

H -2.147872 32.169689 32.637987

H 0.695622 25.525884 26.481859

H 1.040564 27.352735 28.387266

H -1.085017 27.755291 29.771496

H -4.086728 25.361226 28.592242

H -5.116017 26.746359 30.278111

H -4.105130 29.551527 29.345342

H -2.643899 26.757253 32.293158

H -1.692452 28.621618 33.605879

H -3.175408 31.433498 30.654825

H -2.773791 30.903212 34.824040

H -1.106939 30.269677 34.706479

H -2.994623 33.409464 35.825578

H -1.453125 35.274214 36.725402

H 1.108185 34.856979 36.143759

Structure of the complex between compound **15** and the active site of BChE

C 131.684140 107.323830 36.342818

C 128.657916 114.946026 36.299113

C 135.264096 122.867953 34.497037

C 136.907018 116.636020 36.816991

C 140.436088 115.676781 37.886038

C 127.434879 125.135045 34.162977

C 136.138964 124.430020 40.255986

C 138.358992 121.573978 41.397060

C 140.744044 120.716047 38.531965

C 141.531042 110.980993 43.741057

C 144.988880 112.005130 38.319973

C 135.176009 118.502987 50.526018

C 135.416360 110.331222 47.652975

C 134.581622 105.274944 45.791964

C 132.702948 118.950026 46.906030

C 131.025901 121.613474 33.922744

C 131.250945 122.661517 34.831431

C 130.521984 123.837235 34.676159

C 129.587384 123.972774 33.633066

C 129.383129 122.908348 32.743139

C 130.099892 121.716729 32.877219

C 128.799375 125.245972 33.482934

O 131.788178 120.484635 34.128525

C 134.766230 121.857358 33.464939

C 134.599084 122.491607 32.111656

C 135.587970 122.332808 31.125163

C 135.446664 122.922100 29.869462

C 134.293255 123.679952 29.617820

C 133.290342 123.858969 30.579046

C 133.456407 123.256629 31.827791

O 134.214997 124.235223 28.356159

C 130.421484 119.607074 37.011930

C 130.233207 118.237823 37.045695

C 129.367921 117.694968 38.015332

C 128.712093 118.578385 38.939296

C 128.908660 119.976148 38.901050

C 129.764687 120.467341 37.933251

N 127.919596 117.794312 39.788041

C 128.075227 116.440823 39.416767

C 128.948018 116.352427 38.340950

C 129.379365 115.115733 37.637541

O 137.170312 122.620895 39.013163

C 137.014269 123.224825 40.069540

N 137.735813 122.887163 41.219422

C 137.391524 120.412703 41.026145

O 137.825310 119.229385 41.688968

C 134.732037 124.217642 39.677473

C 133.880528 123.253087 40.501727

C 133.965509 121.818583 39.952880

O 134.391154 120.928928 40.731440

O 133.587079 121.613738 38.778072

C 139.697595 121.415713 40.660939

N 139.653809 121.370119 39.283861

C 140.332422 119.321519 38.065197

O 140.750489 121.306457 41.287620

N 136.475625 119.461352 44.345956

C 135.561279 118.535936 44.851334

C 135.068165 118.982145 46.085967

N 135.689701 120.212064 46.337407

C 136.540432 120.468606 45.258528

C 134.105318 118.356875 47.027790

O 135.322478 121.375531 48.772570

C 136.337838 121.182761 49.507507

O 137.509823 121.267237 49.084084

C 136.091548 120.836216 50.975096

C 136.265613 119.330416 51.203333

N 139.003935 116.013366 37.866939

C 138.354236 116.306058 36.679775

O 138.955326 116.278818 35.603937

C 135.893443 114.012268 44.191448

C 135.938423 114.423419 45.526371

C 136.183919 113.492139 46.539381

C 136.392157 112.141354 46.220431

C 136.344789 111.733130 44.877822

C 136.095275 112.666711 43.869091

C 136.667310 111.136780 47.307865

C 140.102344 112.463813 45.253508

C 140.020112 113.696287 44.344808

C 141.444324 111.719794 45.074055

C 139.915759 112.870059 46.722314

C 134.619836 108.497075 44.521610

C 134.956469 107.176288 44.198142

C 134.695566 106.693282 42.901367

C 134.108607 107.510566 41.940369

C 133.783092 108.831551 42.295543

C 134.032434 109.341139 43.575389

C 135.589457 106.273032 45.221986

O 133.206555 109.580626 41.295760

O 130.512185 104.329135 36.185221

C 131.664085 104.819148 36.092895

O 132.695252 104.261995 36.542323

C 131.824956 106.162917 35.366588

C 142.951835 112.838521 39.559761

C 144.251271 113.242250 38.850973

C 143.962253 114.231771 37.714404

H 132.010535 118.475480 47.610021

H 132.296175 118.816556 45.896206

H 132.700387 120.026986 47.115019

H 134.474548 118.480530 48.080990

H 134.069909 117.260618 46.852296

H 135.563825 120.801216 47.209170

H 135.321472 117.634205 44.321629

H 137.158394 121.350707 45.181616

H 135.375903 117.430309 50.611545

H 135.095285 118.741589 49.448627

H 134.191134 118.696185 50.964650

H 137.262140 119.014415 50.833556

H 136.269249 119.125497 52.291902

H 135.078995 121.161053 51.272144

H 136.799940 121.401476 51.607205

H 128.987742 114.036581 35.784625

H 128.850266 115.794790 35.632250

H 127.572957 114.876004 36.432908

H 130.479259 115.146672 37.468007

H 129.204726 114.224327 38.275068

H 127.559593 115.655934 39.941451

H 127.362179 118.127390 40.553192

H 130.747339 117.576311 36.348407

H 128.410121 120.626702 39.611989

H 131.094871 120.047268 36.272587

H 129.955389 121.538961 37.868007

H 135.606173 109.634361 48.476792

H 135.068020 109.736769 46.790044

H 134.587362 110.982236 47.954580

H 137.050263 111.643408 48.218141

H 137.484452 110.453633 46.993872

H 136.215366 113.819179 47.577229

H 136.503688 110.686878 44.620572

H 135.784317 115.472848 45.778985

H 136.060987 112.345518 42.828329

H 135.698034 114.742420 43.397395

H 136.445589 116.833008 35.836244

H 136.726556 117.535517 37.429647

H 136.319714 115.826444 37.280469

H 138.530763 116.046462 38.766897

H 140.991194 116.355644 38.556876

H 140.878558 115.760855 36.875148

H 140.591817 114.640826 38.236596

H 142.486581 110.455358 43.638918

H 141.443638 111.669365 42.891673

H 140.733198 110.236926 43.637237

H 141.580147 110.998711 45.903208

H 142.282886 112.436228 45.163283

H 139.270118 111.768228 44.977399

H 138.951968 113.373239 46.869735

H 140.697614 113.559959 47.055406

H 139.932974 112.001241 47.387212

H 139.044531 114.188955 44.430532

H 140.158908 113.427891 43.292140

H 140.783489 114.439840 44.595816

H 145.930664 112.279413 37.834070

H 144.387127 111.462829 37.582755

H 145.224769 111.304215 39.127221

H 144.913372 113.750095 39.597062

H 144.882275 114.541747 37.208597

H 143.471473 115.136764 38.088458

H 143.303507 113.795793 36.955482

H 143.143693 112.147630 40.387291

H 142.254279 112.342504 38.876252

H 142.435183 113.711069 39.974354

H 131.801549 108.287046 35.834289

H 132.439677 107.276118 37.136225

H 130.700750 107.324028 36.827885

H 131.067947 106.236661 34.565968

H 132.810572 106.192827 34.869345

H 135.047150 104.622702 46.539004

H 134.162440 104.633035 45.007683

H 133.740619 105.785394 46.275890

H 136.442871 105.727533 44.766701

H 136.033753 106.867843 46.046442

H 134.818012 108.885661 45.530642

H 134.954847 105.664643 42.645656

H 133.781289 110.368014 43.829774

H 133.900166 107.151150 40.934468

H 132.974494 110.497754 41.599321

H 135.386420 122.398045 35.481273

H 134.562008 123.700680 34.619330

H 136.234027 123.291915 34.214095

H 135.467191 120.999037 33.412996

H 133.799568 121.418360 33.804775

H 136.479646 121.742280 31.342138

H 132.686004 123.383387 32.592407

H 136.203389 122.805256 29.098321

H 132.404659 124.449365 30.362376

H 133.390951 124.774186 28.240516

H 126.862710 126.063619 34.061360

H 127.537355 124.925874 35.234949

H 126.834833 124.324952 33.732086

H 129.361570 126.103105 33.908024

H 128.666675 125.490842 32.408800

H 130.678098 124.660219 35.375376

H 128.656020 123.006331 31.936575

H 131.978574 122.540076 35.633247

H 129.940379 120.892945 32.186975

H 131.560171 119.757862 33.489286

H 136.055461 124.744804 41.317645

H 134.226568 125.201923 39.616188

H 134.807888 123.850915 38.630822

H 134.170714 123.262440 41.566165

H 132.820084 123.563810 40.478710

H 137.559144 123.390763 42.093510

H 138.606159 121.455842 42.497475

H 136.338469 120.655875 41.299725

H 137.416154 120.159060 39.951404

H 137.467441 119.214958 42.632395

H 138.755382 121.503052 38.787860

H 141.659308 120.659530 39.167782

H 140.082791 118.665501 38.908999

H 141.142166 118.841053 37.502926

H 139.453653 119.347404 37.408298

H 136.619099 125.294322 39.741381

H 141.001172 121.357009 37.656870

S 131.449503 114.857111 41.599110

C 132.832978 115.853904 42.011330

C 132.463185 116.810534 42.905081

C 131.062012 116.749649 43.269851

C 130.381582 115.748054 42.656186

C 134.161049 115.578642 41.403079

N 134.727918 116.740452 40.671207

C 134.245979 117.030935 39.366558

C 133.828556 116.043041 38.484123

C 133.504696 116.378451 37.142585

C 133.611849 117.727386 36.699107

C 134.003424 118.729540 37.645083

C 134.311746 118.398540 38.934945

C 133.315583 118.077290 35.342663

C 132.907627 117.131556 34.431318

C 132.777965 115.800983 34.880641

C 133.059951 115.401905 36.189421

C 132.835768 113.980356 36.402139

C 132.393336 113.327932 35.303093

S 132.228043 114.429396 33.941697

H 134.812324 117.569443 41.276169

H 132.148674 112.285114 35.187292

H 133.015395 113.522597 37.369036

H 132.691996 117.385709 33.396629

H 133.415354 119.126734 35.047342

H 134.026520 119.782978 37.338595

H 134.571073 119.197713 39.655337

H 133.744486 115.006918 38.802761

H 134.135810 114.688758 40.727466

H 134.899603 115.312414 42.217950

H 133.121223 117.563012 43.325337

H 130.634638 117.456483 43.971295

H 129.343363 115.478696 42.749611

**6) Calculated UV-Vis spectra of compounds 2-15**


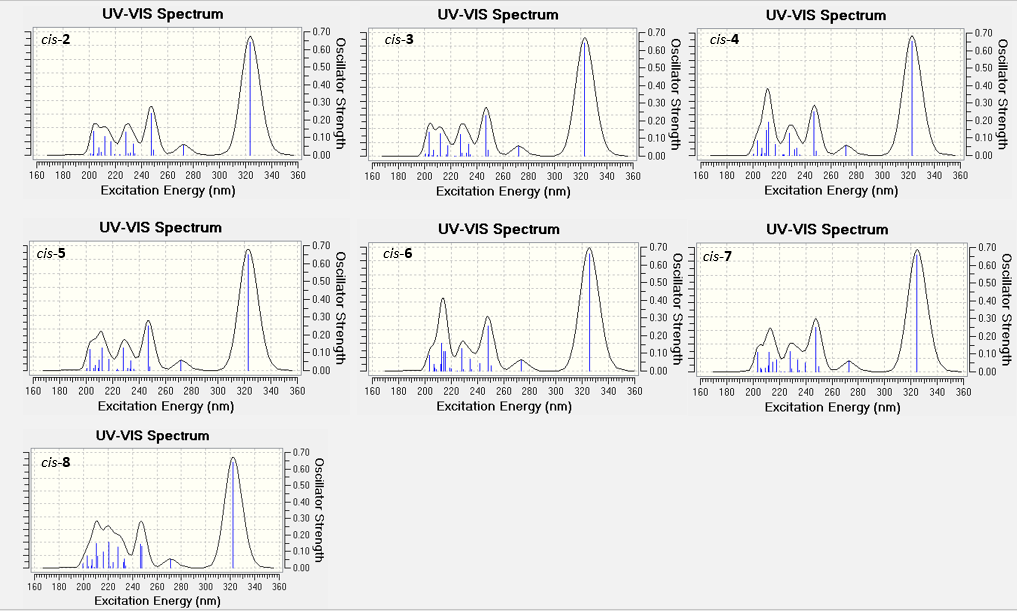


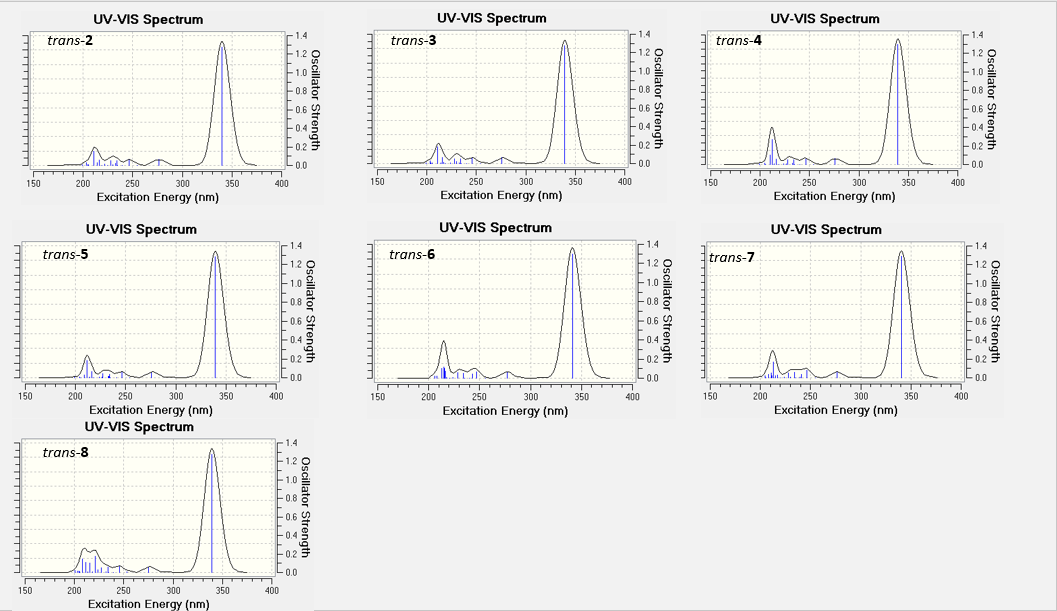


**Table S1.** Measured and calculated values of *λ*_max_ for naphtho-thiophene benzylamines, with main assignments. Calculated data is obtained at the computational model (CPCM)TD-CAM-B3LYP/6–31++ G(d) (ACN, with geometries optimized at M06-2X/6-31G(d) level of theory. The highest occupied and lowest unoccupied molecular orbital are denoted by H and L, respectively.

| **Compound** | ***λ*^exp^/nm** | ***λ*^calc^/nm** | **Assignment** |
| --- | --- | --- | --- |
| **9** | 313, 302 | 310, 238, 220 | H → L, H−1 → L, H−2 → L |
| **10** | 312, 303 | 309, 238, 221 | H → L, H−1 → L, H−2 → L |
| **11** | 256, 245 | 309, 239, 221 | H → L, H−1 → L, H−2 → L |
| **12** | 258, 245 | 309, 239, 221 | H → L, H−1 → L, H−2 → L |
| **13** | 315, 259, 244 | 310, 238, 220 | H → L, H−1 → L, H−2 → L |
| **14** | 314, 279 | 310, 237, 220 | H → L, H−1 → L, H−2 → L |
| **15** | 310, 256, 245 | 307, 238, 220 | H → L, H−1 → L, H−2 → L |


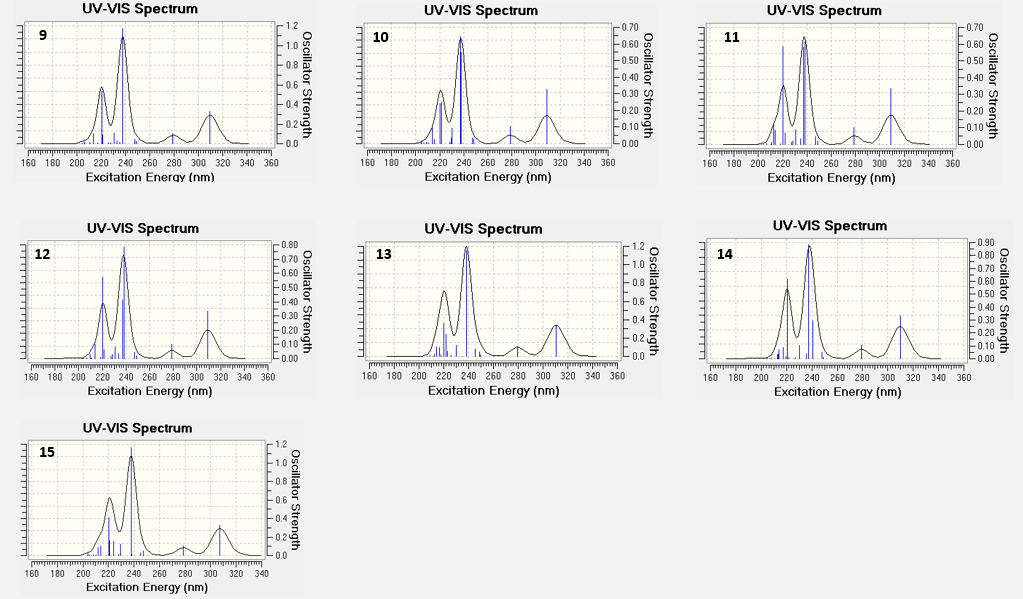

Supplement: Supplementary file 1 [file ijms-24-00610-s001.zip › ijms-2094879-supplementary.docx]
